# A Facile Method to Quantify Synthetic Peptide Concentrations on Biomaterials

**DOI:** 10.1021/acsami.4c07164

**Published:** 2024-09-09

**Authors:** Jonathan P. Wojciechowski, Thomas Benge, Kaili Chen, Cécile Echalier, Ruoxiao Xie, Molly M. Stevens

**Affiliations:** † Department of Materials, Department of Bioengineering and Institute of Biomedical Engineering, Imperial College London, London, SW7 2AZ, United Kingdom; ‡ Kavli Institute for Nanoscience Discovery, Department of Physiology, Anatomy and Genetics, Department of Engineering Science, University of Oxford, Oxford, OX1 3QU, United Kingdom

**Keywords:** peptides, biomaterials, hydrogels, nanoparticles, surfaces, quantification

## Abstract

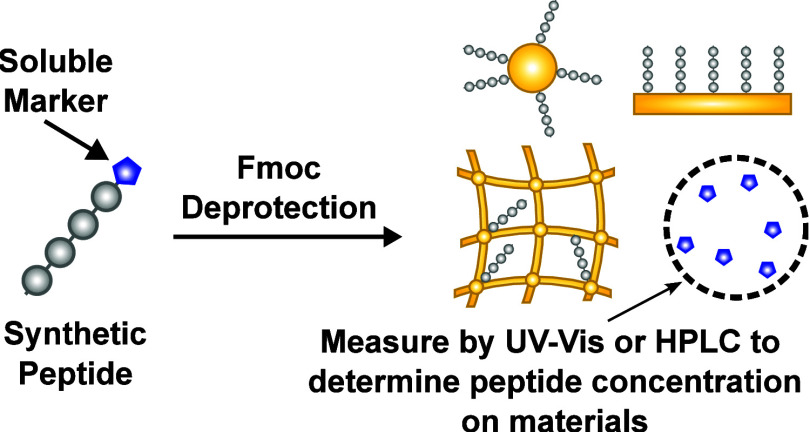

While it is well understood that peptides can greatly improve cell–material interactions, it is often challenging to determine the concentration of the peptide which decorates a material. Herein, we describe a straightforward method using readily, synthetically accessible Fmoc peptides and commercially available reagents to measure the concentration of peptides on nanoparticles, surfaces, and hydrogels. To achieve this, the Fmoc protecting group from immobilized peptides is removed under optimized basic conditions. The dibenzofulvene released can be quantified by HPLC or UV–vis spectroscopy, enabling a direct experimental measurement of the concentration of the peptide. We show that we can measure the concentration of a BMP-2 peptide mimic on a hydrogel to determine the concentration required to stimulate osteogenesis of human mesenchymal stem cells. We envision that this methodology will enable a more thorough understanding of the concentration of synthetic peptides decorated on many biomaterials (e.g., nanoparticles, surfaces, hydrogels) to improve deconvolution of the interactions at the cell–material interface.

## 1 Introduction

Synthetic peptides are molecules that are highly utilized to improve the bioactivity of biomaterials. The ability of peptides to mimic the functions of larger proteins, their easily manipulatable chemistry, and their low-cost synthesis make peptides ideal compounds to decorate a range of materials such as nanoparticles, 2D surfaces, and 3D materials, like hydrogels. Within these applications, peptides are typically immobilized to a surface or within a matrix using covalent chemistry to improve their bioavailability; however, a key challenge is quantifying to what extent these peptides have modified a material. Ultimately, the effectiveness of a biomaterial is largely dictated by its cell–material interactions,^1^ hence it is crucial not only to deconvolute these interactions but also to thoroughly characterize materials for tissue engineering or translational applications.^2^

Two strategies are used to quantify peptide concentrations on materials, namely, measuring the peptide concentration directly on the material or inferring the concentration through an indicator or marker in solution. Methods such as time-of-flight selective ion mode mass spectrometry (ToF-SIMS),^3^ solid-state fluorescence spectroscopy,^4^ and X-ray photoelectron spectroscopy (XPS) can measure the presence of peptides directly on materials (and in some cases quantify);^5,6^ however, they all require calibration with each measurement, specialist instrumentation and are material-dependent. Non-natural fluorinated amino acids have also been used as a marker to quantify peptide concentrations on silica nanoparticles by ^19^F NMR; however, this method requires the nanoparticles to be dissolved.^7^ An alternative approach is to measure the peptide concentration indirectly using a reagent that interacts or reacts with the peptide to determine its concentration. Peptide quantification methods exist which target functional groups common to peptides such as amines (*e*.*g*. ninhydrin,^8^ fluorescamine,^9^ trinitrobenzenesulfonic acid (TNBS),^10^ and fluoraldehyde^11^ assays), thiols (Ellman’s assay^12^), and amide bonds (Biuret test,^13^*i*.*e*., bicinchoninic acid assay (BCA assay)). Unfortunately, they all suffer drawbacks such as sensitivity, long reaction time frames, or the requirement for specific functional groups which are often instead used to covalently modify biomaterials, ultimately limiting their standardized usage. While these assays can provide quantification of the peptides on materials if a single functional group is available, multiple functional groups are common on longer peptides and can cause non-linear responses, making results difficult to interpret via a standard curve. Additionally, most of these reagents react with the peptide to produce an insoluble product which cannot be quantified on a material. The exceptions are the ninhydrin test, which instead requires heating (>70 °C) that is largely incompatible with common biomaterials (*e*.*g*. poly(esters), low glass transition (*T*_g_) polymers, nanoparticles with heat instability), picric acid titration,^14^ which uses high risk, potentially explosive reagents, and the Ellman’s assay, which requires a thiol functional group and suffers from high background noise when performed over longer incubation periods required for completion. As such, accessible methods provide only a qualitative measure of peptide functionalization on a material or require specialist techniques and instrumentation.

We reasoned that a method with fast kinetics to produce a soluble product capable of being measured in solution would be ideal for the quantification of peptide concentrations on biomaterials. In our search for a suitable method to characterize peptide concentrations on biomaterials we were interested in utilizing the fluorenyl methoxy carbonyl (Fmoc) group which is commonly used in Fmoc solid phase peptide synthesis as a protecting group for primary amines.^15,16^ Additionally, Fmoc protected amino acids are widely available commercially and commonly used in peptide synthesis to quantify amino acid loading on solid supports and to monitor reaction completion in peptide synthesis. In other cases, Fmoc peptides have been used as Raman reporters for measuring enzyme activity^17^ and to monitor peptide synthesis on dynamic surfaces for mesenchymal stem cell growth.^3^ Fmoc deprotections have also been used to measure the concentration of amines on mesoporous silica;^18^ however, to the best of our knowledge they have not been demonstrated as a general strategy for quantifying peptide concentrations on biomaterials.

**Figure 1:**
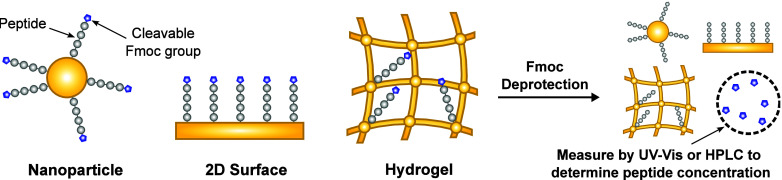
Assay principle to determine peptide concentrations on a range of materials. A cleavable Fmoc group is left on the peptide synthesized via Fmoc solid phase peptide synthesis to act as a soluble marker to determine the peptide concentration. Due to the stoichiometry, the concentration of the cleaved dibenzofulvene from the Fmoc group, which can be measured in solution, is equivalent to the concentration of the peptide on the material.

Herein, we describe a straightforward method for determining synthetic peptide concentrations on a variety of biomaterials. Using commercially available Fmoc amino acids, we show that Fmoc-protected peptides are convenient and accessible derivatives to quantify the concentration of a peptide immobilized on a biomaterial. We utilize the base catalyzed deprotection of the Fmoc group to produce dibenzofulvene (DBF) as a stoichiometric marker to infer the concentration of peptide immobilized on a material, while leaving the native peptide on the biomaterial of interest (Figure 1). In contrast to conventional Fmoc deprotection conditions (*i*.*e*., 20% (v/v) piperidine in *N*,*N*-dimethylformamide (DMF)), we show that 0.25 M sodium hydroxide in 1:1 (v/v) methanol/water can be used as a cleavage reagent to maximize biomaterial compatibility. This cleavage is quantitative and gives a soluble dibenzofulvene (DBF) product, which can be measured via UV–vis or HPLC. Because the released dibenzofulvene is in a 1:1 stoichiometry with the peptide, it can be used as a soluble marker to measure the concentration of the peptide on the biomaterial.

## 2 Results and Discussion

### 2.1 Determining Optimized Fmoc Cleavage Conditions

We were initially motivated to use the Fmoc protecting group due to its established usage in Fmoc solid phase peptide synthesis (SPPS) to determine resin loading and its wide commercial availability. Typically in Fmoc SPPS, the Fmoc group is removed using an appropriate base (*e*.*g*., piperidine in *N*,*N*-dimethylformamide (DMF)), however, with consideration that most biomaterials are used in aqueous environments (and eventually with cells), we were keen to omit the usage of cell incompatible reagents which may be present in trace amounts and be difficult to remove. Additionally, biomaterials based on ester or amide linkages are largely incompatible with DMF due to their solubility in the solvent. We decided on sodium hydroxide as an appropriate base for the Fmoc cleavage, as it has been shown to be suitable for Fmoc deprotections in a mixture of 1:1 (v/v) 2-methyltetrahydrofuran/methanol.^19^ We used Fmoc-Arg(Pbf)-OH as a model Fmoc-amino acid, as the Pbf protecting group would allow for easy monitoring via HPLC. We anticipated conducting the Fmoc deprotection under completely aqueous conditions. Unfortunately, due to the poor solubility of Fmoc-Arg(Pbf)-OH in 0.25 M aqueous sodium hydroxide solutions, methanol was added as a co-solvent. Initially, we screened varying volume ratios of methanol (10–50%, v/v) in 0.25 M aqueous sodium hydroxide solutions using Fmoc-Arg(Pbf)-OH at a final concentration of 1 mM. The Fmoc-Arg(Pbf)-OH was initially soluble in all mixtures, but upon addition of the aqueous sodium hydroxide solution, a white precipitate formed (likely dibenzofulvene, DBF) when the methanol concentration was below 50% (v/v). In contrast, all products remained soluble in the 50% (v/v) methanol mixture, and hence that was used as the minimum methanol volume required (Figure S1).

**Figure 2:**
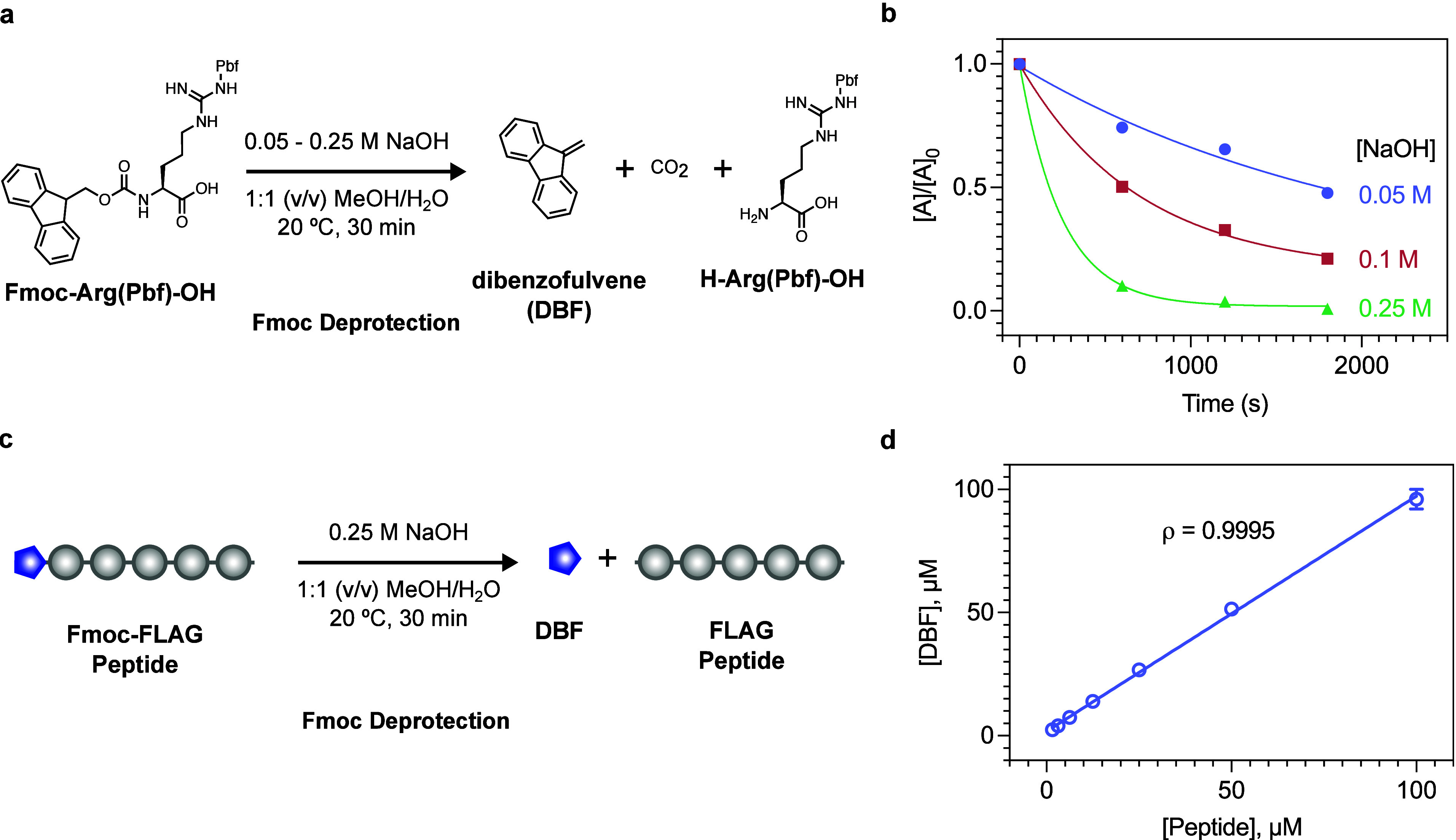
(a) Reaction scheme using Fmoc-Arg(Pbf)-OH to determine the optimal concentration of sodium hydroxide (NaOH) required for Fmoc deprotection. (b) HPLC kinetics of the Fmoc deprotection of Fmoc-Arg(Pbf)-OH with varying NaOH concentration, where [A] is the concentration of Fmoc-Arg(Pbf)-OH with respect to time and [A]_0_ is the initial concentration of Fmoc-Arg(Pbf)-OH. (c) Extending the Fmoc deprotection conditions to a model peptide (FLAG, Fmoc-DYKDDDDKGGGGC) shows an excellent correlation (ρ = 0.995) between the concentration of the peptide and the measured concentration of dibenzofulvene (DBF). Data shown as mean ± s.d. *n* = 3.

Next, we sought to determine the optimal sodium hydroxide concentration for the assay. Considering that many biomaterials contain ester linkages, we had to strike a balance between preventing potential hydrolysis of these ester linkages if the hydroxide concentration was too high, while allowing for the assay to be conducted in an appropriate time frame (arbitrarily set to 30 min). We screened three concentrations of aqueous sodium hydroxide solution in 1:1 (v/v) methanol/water and monitored the Fmoc deprotection kinetics using HPLC (Figure 2a,b). The HPLC chromatograms (Figure S2) show the disappearance of the peak associated with Fmoc-Arg(Pbf)-OH at *t*_R_ = 5.59 min, and the appearance of two peaks at *t*_R_ = 4.08 min (assigned to H-Arg(Pbf)-OH) and *t*_R_ = 5.82 min (assigned to DBF). We assigned the HPLC peaks based on the ESI-MS spectrum for H-Arg(Pbf)-OH (Figure S3). For DBF, since a mass ion could not be found via ESI-MS, we assigned based on comparison of the retention time and UV–vis spectra of a DBF sample purified using preparative-HPLC against a commercial standard (Figure S4). The kinetics show that, while the reaction proceeds at 0.05 and 0.1 M, assuming first-order reaction kinetics, it would take approximately 166 and 56 min, respectively, for completion (*i*.*e*. 7 × *t*_1/2_). In contrast, at 0.25 M, the deprotection of Fmoc-Arg(Pbf)-OH is complete within approximately 20 min and hence is a suitable concentration for a reaction time of 30 min or less.

Typically, in Fmoc SPPS, the piperidine acts as both a base to deprotect the Fmoc group and as a scavenger to react with the generated dibenzofulvene to avoid a potential reaction with the liberated α-amino group of the amino acid, which would otherwise react and irreversibly modify the growing peptide chain. We therefore screened a variety of scavengers in excess to Fmoc-Arg(Pbf)-OH (100 mol equiv) under these cleavage conditions; however, to our surprise we found none to be particularly reactive with the generated DBF even after reaction for 24 h (Table S1). We can therefore assume that reaction of the generated DBF with the liberated α-amino group of H-Arg(Pbf)-OH is negligible under these conditions. Since this reaction typically proceeds well in DMF, we assume that the solvent conditions used for the cleavage make this reaction less prominent. Favorably, it has been shown that the determination of amino acid loading through UV–vis analysis of DBF as opposed to the DBF-piperidine adduct results in a more accurate result due to errors which occur based on the differing extinction coefficients of the two derivatives.^20^ Additionally, when using poly(ethylene glycol) based resins such as ChemMatrix or Tentagel, we show that these Fmoc cleavage conditions are suitable for determining Fmoc amino acid loading via UV–vis (Figure S5 and Table S2). Knowing the liberated DBF can be used as a soluble marker, we wanted to verify the validity of this method by comparing known concentrations of a model peptide to the measured concentration of DBF as determined by HPLC. To verify the reaction conditions, we synthesized a model Fmoc protected FLAG-tag peptide, a peptide tag commonly used in recombinant protein synthesis to aid in purification (Fmoc-DYKDDDDKGGGGC). We reacted the Fmoc-FLAG peptide in 0.25 M aqueous sodium hydroxide in 1:1 (v/v) methanol/water for 30 min, then determined the concentration of Fmoc-FLAG peptide using a DBF standard curve via HPLC and compared this with the known concentration of the Fmoc FLAG peptide. The analysis showed an excellent correlation (ρ = 0.995) between the known concentration of peptide versus the concentration of DBF determined from a standard curve (Figure 2c,d). We therefore chose 0.25 M aqueous sodium hydroxide in 1:1 (v/v) methanol/water for 30 min as the optimized cleavage conditions.

Additionally, we were interested in testing the stability of common poly(esters) such as poly(caprolactone) (PCL) and poly(lactic acid-*co*-glycolic acid) (PLGA) under the Fmoc cleavage conditions. Slower degrading poly(esters) such as PCL showed no significant change in molecular weight by gel permeation chromatography (GPC), whereas faster degrading poly(esters) such as PLGA showed a decrease in molecular weight (Figure S6). While this method is unsuitable for faster degrading poly(esters) (*e*.*g*. poly(lactic acid), poly(glycolic acid), poly(anhydrides)), it can be suitable for slower degrading poly(esters) on a case by case basis.

### 2.2 Fmoc Deprotections to Quantify Peptides on a Model Surface

**Figure 3:**
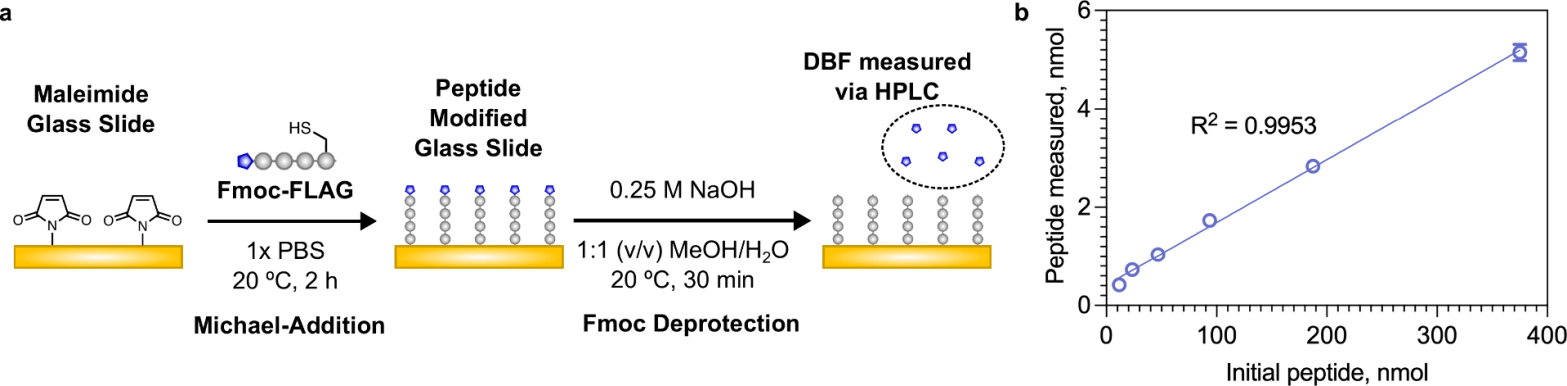
(a) Reaction scheme using an Fmoc-FLAG peptide to modify a maleimide functionalized glass surface using Michael addition, followed by Fmoc deprotection to determine the concentration of the peptide on the surface. (b) Concentration of FLAG peptide measured on the surface by the Fmoc assay versus the concentration of peptide used for the reaction. Data shown as mean ± s.d. *n* = 3.

With the reaction conditions optimized, we sought to use the assay to determine the concentration of peptides conjugated on a variety of biomaterials. Commonly, surfaces are modified with peptides to improve adhesion with cells via their integrin receptors. We utilized maleimide modified glass as our model surface, which was functionalized with the Fmoc-FLAG peptide via a thiol-maleimide Michael addition reaction (Figure 3a). We placed the Fmoc-peptide modified glass under the Fmoc deprotection conditions for 30 min, then measured the concentration of DBF in the supernatant via HPLC (Figure 3b). We observed a linear trend in the concentration of peptide used for functionalization compared with the concentration of DBF measured (and hence concentration of peptide on the surface). Since the peptide is almost always used in excess to drive the reaction to completion, it is difficult to infer the concentration of the peptide conjugated on the surface by analyzing unconjugated peptide in the supernatant. Interestingly, the concentration used in the reaction is almost 2 orders of magnitude higher than the concentration immobilized on the surface, highlighting the need to characterize the concentration of the peptide on the surface directly.

### 2.3 Fmoc Deprotections to Quantify Peptides on a Model Nanoparticle

**Figure 4:**
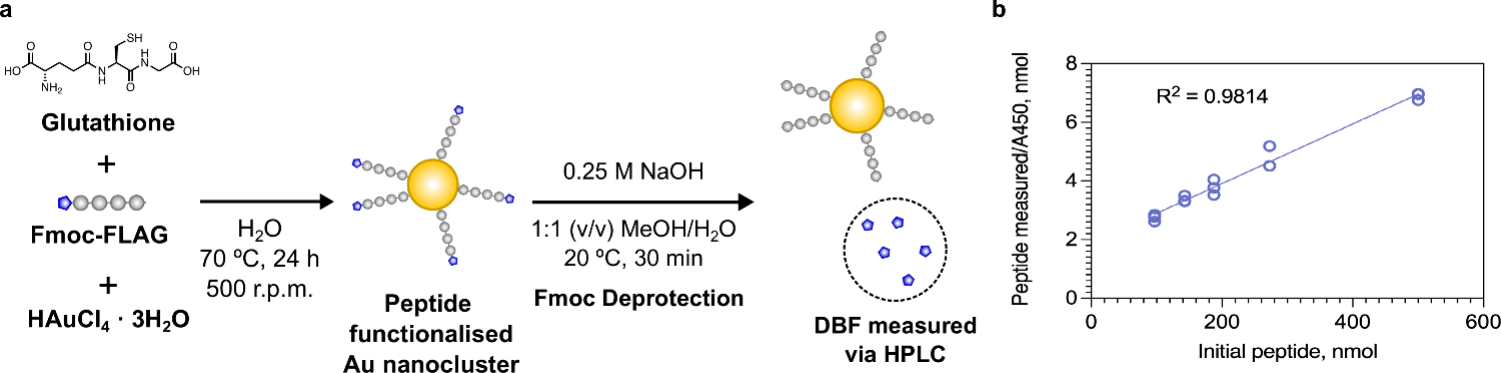
(a) Reaction scheme to synthesize peptide conjugated gold nanoclusters (AuNC) from glutathione (GSH), Fmoc-FLAG peptide, and gold(III) chloride trihydrate. (b) Measured concentration of the peptide from the Fmoc assay normalized to A450 versus the initial Fmoc-FLAG peptide concentration. Data shown as individual repeats. *n* = 2/3.

We next sought to demonstrate the assay on another common biomaterial, nanoparticles, which are often modified with peptides in a therapeutic or imaging context to improve targeting toward a specific cell or tissue. Previously, we described the use of Au nanoclusters to measure MMP activity for *in vivo* disease monitoring.^21^ As a demonstration, we synthesized Au nanoclusters in the presence of the model Fmoc-FLAG peptide. We verified that, under the reaction conditions, the Fmoc group remains stable (Figure S7). We employed the assay conditions, pelleted the particles by centrifugation, and measured the concentration of DBF in the supernatant via HPLC (Figure 4a). To account for differences in the concentration of particles between samples due to losses in the purification, we normalized the nanomoles of peptide, determined from the concentration of cleaved DBF to the absorbance of the particle solution at 450 nm, which can be used as a proxy for nanoparticle concentration. We observed a linear trend between the initial peptide concentration used and the nanomoles of peptides measured per particle based on the concentration of DBF, which allowed us to estimate the number of peptides per particle (Figure 4b).

### 2.4 Fmoc Deprotections to Quantify Peptides on a Model Hydrogel

**Figure 5:**
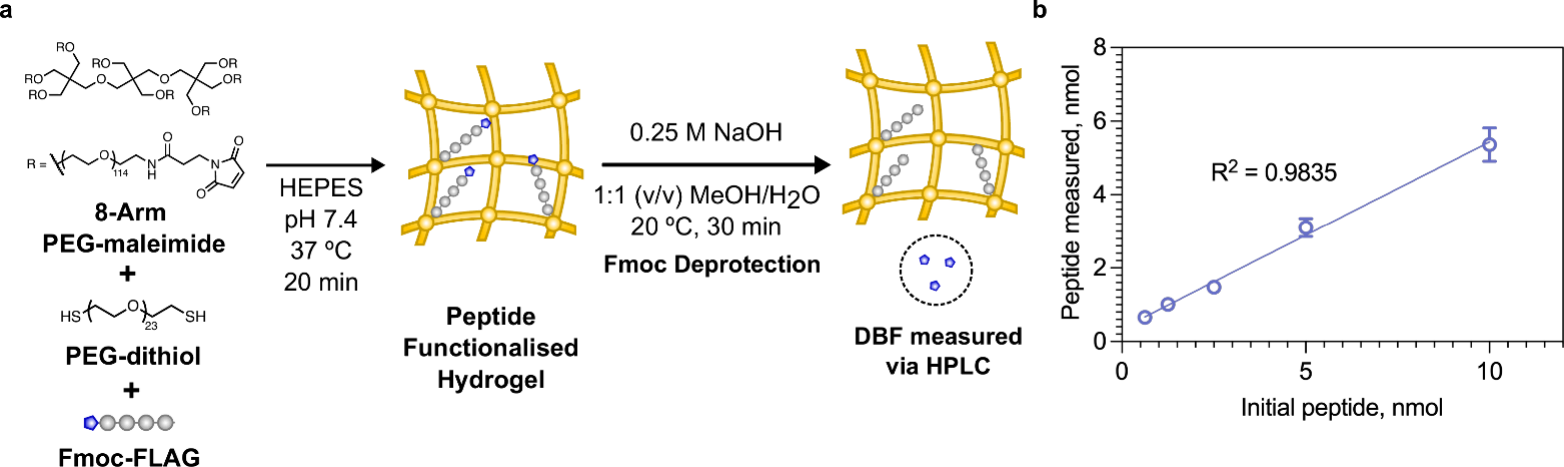
(a) Fabrication of PEG-hydrogels from a 40 kDa 8-arm PEG-maleimide functionalized with Fmoc-FLAG peptide and cross-linked with 1 kDa PEG-dithiol. (b) Measured concentration of peptide into the hydrogel determined using the Fmoc assay versus the initial concentration of peptide used. Data shown as mean ± s.d. *n* = 3.

Some of the most widely utilized biomaterials are hydrogels. We selected 8-arm PEG macromers modified with maleimides which could be cross-linked with a PEG-dithiol to create hydrogels. These materials are often used to investigate the effect of the 3D viscoelastic environment on cells. The 8-arm PEG macromers were first modified using Michael addition with the Fmoc-FLAG peptide, followed by cross-linking via Michael addition with a 1 kDa PEG-dithiol to make the hydrogels. The hydrogels were allowed to equilibrate for 24 h, washed with HEPES buffer, and then placed in the Fmoc deprotection solution (Figure 5a). Due to the porosity of the hydrogels, there is a delay in the release of the DBF from the hydrogel network. We showed that this delay was independent of the NaOH concentration, and at 9 h all DBF was released from the hydrogels, as shown by a plateau in DBF concentration with respect to time (Figure S8). We again observed a linear trend between the concentration of peptide initially reacted with the 8-arm PEG-maleimide and the concentration of DBF measured via HPLC. Interestingly, even though cysteine maleimide Michael additions are very efficient reactions, after 2 h of reaction at 37 °C, a commonly used reaction time, the concentration range tested resulted in a range of conjugation efficiencies (Figure 5b). This highlights the need to characterize the peptide concentration on biomaterials, as the density of peptide grafted throughout the biomaterial can have significant effects on its bioactivity.

### 2.5 Quantifying the Concentration of a BMP-2 Peptide for Bone Tissue Engineering

**Figure 6:**
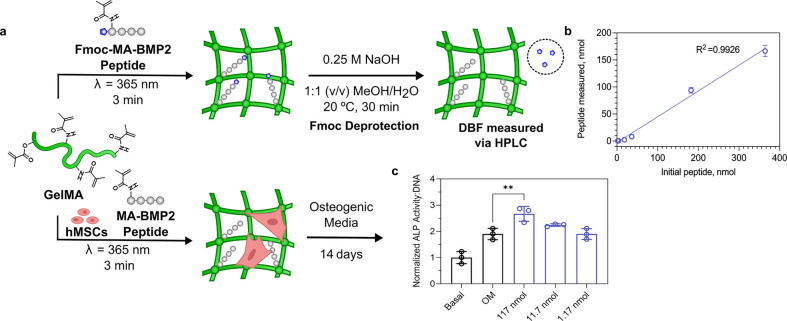
(a) Method to functionalize GelMA hydrogels with the Fmoc-MA-BMP2 peptide and measure the concentration of MA-BMP2 peptide. (b) Measured concentration of MA-BMP2 peptide on the hydrogels compared with the initial Fmoc-MA-BMP2 peptide concentration used. Data shown as mean ± s.d. *n* = 3. (c) Measured ALP activity in basal, osteogenic media, or osteogenic media with MA-BMP2 peptide (normalized to DNA content). Data shown as mean ± s.d. *n* = 3, **p < 0.005, ordinary one-way ANOVA followed by Tukey’s post hoc test.

We have demonstrated that the Fmoc assay is a useful method to determine peptide concentration on a range of biomaterials (surfaces, nanoparticles, and hydrogels). We wanted to apply this method in a tissue engineering context. Bone morphogenetic protein 2 (BMP2) is a growth factor that is commonly used to promote osteogenesis of human mesenchymal stem cells. The structure of BMP2 is as a homodimer which binds to BMP receptor type 1 via the “wrist” epitope and the type 2 receptor via the “knuckle” epitope. A peptide sequence derived from the “knuckle” epitope of BMP2 was reported by Saito et al. to act as a mimic for the “knuckle” epitope of BMP2, inhibiting binding of BMP2 to BMP receptor types 1 and 2 in competition assays and promoting ectopic calcification in a rat calf model.^22^ While the ability of the peptide to mimic the “knuckle” epitope of BMP2 as a soluble factor is disputed, the activity has been verified by Madl et al. when covalently immobilized to an alginate hydrogel.^23^ The degree of functionalization of the BMP2 mimicking peptide onto the alginate was well-characterized using ^1^H NMR by the authors; however, one limitation of the approach is that it does not measure the concentration of the peptide in the hydrogels, which could differ based on the cross-linking efficiency and dissolution of BMP2 modified alginates not cross-linked into the hydrogel network. We sought to apply the Fmoc assay described above to determine the concentration of a BMP2 mimicking peptide using GelMA as a model hydrogel (Figure 6a). We synthesized an Fmoc-methacrylamide-BMP2 peptide derivative containing a lysine modified with a methacrylamide group which could be cross-linked into the hydrogel during the free-radical photo-cross-linking of the hydrogel network. We first characterized the GelMA hydrogels containing an increasing concentration of conjugated Fmoc-methacrylamide-BMP2 peptide (Fmoc-MA-BMP2; Figure 6b), showing a linear correlation between the initial amount of peptide added to the hydrogels and the concentration of peptide measured, as inferred by the concentration of DBF by HPLC. We then performed a tissue engineering experiment with human mesenchymal stem cells (hMSCs) encapsulated in GelMA hydrogels containing covalently attached methacrylamide-BMP2 peptides (MA-BMP2) for 14 days. Compared with basal media and osteogenic media controls, the hydrogels in osteogenic media conjugated with 117 nmol of BMP2 peptide showed a statistically significant increase in ALP activity, whereas the lower concentrations of BMP2 (*i*.*e*., 11.7, 1.17 nmol) showed no significant differences from the osteogenic media control (Figure 6c). These results imply that while the BMP2 peptide can increase ALP activity, a relatively high concentration is required. This is consistent with previous reports, where immobilization of the peptide onto a material was required to ellicit an increase in ectopic calcification^22^ or ALP activity.^23^ We speculate that while this BMP2 peptide mimic shows some activity, the binding affinity toward BMP2 receptor type 2 is likely low, hence why no osteogenic effects are seen when the peptide is used as a soluble factor, and only at high concentrations present when immobilized on a material.

## 3 Conclusions

We have described a straightforward method to characterize the concentration of synthetic peptides on a range of biomaterials (surfaces, nanoparticles, and hydrogels). In this method, we utilized commercially available Fmoc-amino acids with optimized Fmoc deprotection conditions to determine the concentration of peptides on biomaterials. Due to the stoichiometry of the Fmoc group on the peptide, the concentration of the peptide on the biomaterial can be calculated by measuring the concentration of dibenzofulvene (DBF) released in solution in the assay, circumventing challenging surface analysis techniques. We show that the concentration of DBF can be measured via HPLC and UV–vis, which we anticipate will enable this assay as an accessible and broadly applicable method. Using this method, we show that the concentration of a BMP2 peptide mimic can be quantified, providing insight into the concentrations required to increase the ALP activity of hMSCs encapsulated within a hydrogel. While the Fmoc group enables quantification of the peptide concentration on a range of biomaterials, the limitations of this approach are that it will increase the hydrophobicity of the peptide. This can be circumvented by engineering hydrophilic amino acids as peptide linkers to counteract the increased hydrophobicity. This strategy increases the water solubility of the N-Fmoc peptide and, advantageously, increases availability of the peptide. Additionally, purification of the N-Fmoc functionalized peptides after Fmoc SPPS should be straightforward as these derivatives are common materials for self-assembled peptide hydrogels which are purified using standard purification methods (i.e., precipitation in cold diethyl ether solution^24^ and/or Prep-HPLC^25^). An additional consideration is that conjugation via the N-terminus of the peptide is not possible. While methods exist to target the α-amino group of a peptide based on p*K*_a_ differences to other amino groups (*e*.*g*., lysine ε-amino), the authors would suggest going with a more selective approach such as incorporating a cysteine (*e*.*g*., for Michael additions), a methacrylamide derivative as shown, or a commercially available biorthogonal chemistry derivative (*e*.*g*., Fmoc-azidohomoalanine for strain-promoted azide alkyne cycloadditions). Ultimately, we hope this accessible method will allow bioengineers, tissue engineers, and biomaterial scientists to precisely characterize peptide concentrations on biomaterials and enable a more thorough understanding of the cell-material interface based on the effects of peptide surface concentrations.

## 4 Experimental Section

### 4.1 Materials

All chemicals were obtained from Sigma-Aldrich and used as received unless otherwise specified. Solvents were obtained from VWR. Side chain protected Fmoc-amino acids were obtained from AGTC Bioproducts Ltd., Fluorochem, or Iris Biotech. The peptide coupling reagent *N*,*N*′-diisopropylcarbodiimide was obtained from Manchester Organics, and hexafluorophosphate azabenzotriazole tetramethyl uranium (HATU) was obtained from Fluorochem. Solid phase peptide synthesis resins were obtained from Sigma-Aldrich (Wang ChemMatrix resin, 0.5–1.2 mmol/g loading, 35–100 mesh particle), Chem Impex (2-chlorotrityl chloride, 1.14 mmol/g loading, 200–400 mesh particle), or Rapp Polymere (TentaGel XV HMPA resin, 0.2–0.4 mmol/g loading, 100–200 μm particle size). Maleimide functionalized glass slides, 25 × 75 mm were obtained from PolyAn (Product Number: 104 00 441). Functionalized 8-arm PEG maleimide (*M*_w_ = 40 kDa) was obtained from Jenkem Technology USA. MesenPro RS Medium (#12746012), Trypsin-EDTA (#25300054), mesenchymal stem cell qualified fetal bovine serum (#12662029), αMEM supplemented with GlutaMAX (#32561037), and 1% penicillin/streptomycin (#15070063) were obtained from Thermo Fisher. β-Glycerophosphate (#G9422), 50 μg/mL l-ascorbic acid 2-phosphate (#G9422), and 100 nM dexamethasone (#D4902) used in cell culture were obtained from Sigma-Aldrich.

### 4.2 Glass Slide Modification with Fmoc-FLAG Peptide

In a typical experiment, a 2 mM stock solution of Fmoc-FLAG peptide was dissolved in 1× PBS. An equal volume aliquot to the Fmoc-FLAG peptide stock solution of TCEP immobilized resin was transferred to an Eppendorf tube and centrifuged at 1000 rcf for 1 min. The supernatant was removed, and then the resin was resuspended in Fmoc-FLAG peptide stock solution and left shaking overnight at room temperature. The resin was then centrifuged at 1000 rcf for 1 min, the supernatant removed, and the concentration checked using an HPLC calibration curve of the Fmoc-FLAG peptide. A dilution series of the Fmoc-FLAG peptide from 500 to 15.625 μM was then prepared. Each maleimide coated glass slide was cut into four equal sized pieces, rinsed with ethanol, dried with nitrogen, and then placed in a six-well plate. A 750 μL aliquot of the Fmoc-FLAG peptide was then added on top of each maleimide glass slide and left for 2 h at room temperature to react. After 2 h, the peptide solution was removed; the disc was washed with DI water (5×), ethanol (2×) and left to dry at room temperature.

### 4.3 Gold Nanocluster Synthesis with Fmoc-FLAG Peptide

In a typical experiment, a 5 mM stock solution of Fmoc-FLAG peptide was prepared in water, with the pH adjusted to 7 with small aliquots of sodium hydroxide solution. An equal volume aliquot to the Fmoc-FLAG peptide stock solution of TCEP immobilized resin was transferred to an Eppendorf tube and centrifuged at 1000 rcf for 1 min. The supernatant was removed, then the resin was resuspended in Fmoc-FLAG peptide stock solution and left shaking overnight at room temperature. The resin was then centrifuged at 1000 rcf for 1 min, the supernatant removed, and the concentration checked using an HPLC calibration curve of the Fmoc-FLAG peptide to prepare a 2 mM stock solution of Fmoc-FLAG peptide. Reactions were prepared where DI water, 20 mM gold(III) chloride trihydrate (HAuCl_4_) in DI water, 20 mM glutathione in DI water, and 2 mM Fmoc-FLAG peptide in DI water were combined as described in Table S3. Each reaction was heated at 70 °C with gentle stirring (500 rpm) for 24 h. The nanoparticles were purified using Amicon Ultra-15 centrifugal filters (10 kDa MWCO) to a final volume of 500 μL.

### 4.4 PEG-Hydrogel Synthesis with Fmoc-FLAG Peptide

A 2.5 mM stock solution of the Fmoc-FLAG peptide was prepared in 20 mM HEPES buffer (pH = 7.4). An equal volume aliquot to the Fmoc-FLAG peptide stock solution of TCEP immobilized resin was transferred to an Eppendorf tube and centrifuged at 1000 rcf for 1 min. The supernatant was removed, and then the resin was resuspended in Fmoc-FLAG peptide stock solution and left shaking for 1 h at room temperature. A 10% (w/v) solution of 8-arm PEG-maleimide (*M*_w_ = 40 kDa) was prepared in HEPES buffer. Then, a 1% (w/v) solution of PEG-dithiol (*M*_w_ = 1 kDa) was prepared in HEPES buffer. The resin was then centrifuged at 1000 rcf for 1 min, the supernatant removed, and the concentration checked using a HPLC calibration curve of the Fmoc-FLAG peptide to prepare a 1 mM stock solution of Fmoc-FLAG peptide. A dilution series of the Fmoc-FLAG peptide from 1 mM to 62.5 μM was then prepared. The 8-arm PEG maleimide stock was mixed in a 1:1 (v/v) ratio with the Fmoc-FLAG peptide and allowed to react at 37 °C for 2 h. Then, 20 μL of the Fmoc-FLAG conjugated 8-arm PEG maleimide solution was mixed with 20 μL of PEG-dithiol solution in a mold made from a 1 mL syringe to form the hydrogels. The plunger was moved up and down to mix the two solutions well, then the syringes were allowed to react at 37 °C for 20 min. After curing, the hydrogels were dispensed into a 96 well plate containing 200 μL of HEPES buffer in each well. The hydrogels were incubated overnight in the fridge to remove any unbound peptide before usage.

### 4.5 General Procedure—Fmoc Deprotection on 2D Surface

A cleavage solution containing 0.25 M sodium hydroxide in 1:1 (v/v) methanol/water was prepared. A 200 μL aliquot of cleavage solution was added to each glass slide, which was then covered at room temperature for 30 min. The cleavage solution was removed, and the concentration of dibenzofulvene was determined from an HPLC calibration curve.

### 4.6 General Procedure—Fmoc Deprotection on a Nanoparticle

A stock solution of 6 M sodium hydroxide in DI water was prepared. Taking into account the volume of water in which the nanoparticle is dispersed, a final concentration of 0.25 M sodium hydroxide in 1:1 (v/v) methanol/water was prepared. The particles were dispersed in 330 μL of water, hence, 360 μL of methanol and 30 μL of 6 M sodium hydroxide solution were added to the nanoparticle suspension. The nanoparticle suspension was left at room temperature for 30 min. A further 360 μL of methanol was added to the suspension to aid in pelleting the nanoparticles, which were centrifuged at 20 000 *g* for 30 min. The supernatant was removed, and the concentration of dibenzofulvene was determined from an HPLC calibration curve.

### 4.7 General Procedure—Fmoc Deprotection on a Hydrogel

A cleavage solution containing 0.25 M sodium hydroxide in 1:1 (v/v) methanol/water was prepared. The HEPES buffer was removed from the hydrogels, and then the hydrogels were washed three times in HEPES buffer and then blotted with a kim wipe to remove excess liquid. The hydrogels were transferred to a 96-well plate, and then to each hydrogel was added 200 μL of the cleavage solution. The reaction was allowed to proceed at room temperature for 4 h, then the supernatant was removed, and the concentration of dibenzofulvene determined from an HPLC calibration curve.

### 4.8 Cell Culture

Human mesenchymal stem cells (hMSCs) were obtained from Lonza (PT-2501). The cells were expanded in T-flasks with the expansion media changed every 2–3 days. The expansion media contained MesenPro RS medium supplemented with 2% (v/v) GlutaMAX solution and 1% (v/v) penicillin/streptomycin. Flasks were grown to 70–80% confluency before being passaged using 0.05% Trypsin-EDTA for 3–4 min at 37 °C to detach cells. All cells used for experimentation were passage 4 or 5, with passage 3 aliquots frozen down and cryo-stored in a 5:4:1 (v/v) solution of MesenPro RS/FBS/DMSO using mesenchymal stem-cell-qualified fetal bovine serum and sterile grade DMSO. Basal media contained αMEM supplemented with GlutaMAX, 10% (v/v) mesenchymal stem cell qualified FBS, and 1% (v/v) penicillin/streptomycin. Osteogenic media contained basal media supplemented with 10 mM β-glycerophosphate, 50 μg/mL l-ascorbic acid 2-phosphate, and 100 nM dexamethasone.

### 4.9 Modification of GelMA Hydrogels with the BMP2 Peptide

A dilution series from 9.1 mM to 45.5 μM of the BMP2 peptide in 1× PBS and a stock solution containing 20 mM lithium phenyl-2,4,6-trimethylbenzoylphosphinate (LAP) in 1× PBS was prepared and sterile filtered. Under sterile culture conditions, the components were mixed as described in Table S4 to make five hydrogels:

The tubes were sealed and heated at 37 °C with vortexing to dissolve the GelMA. A cell suspension of hMSCs (passage 5) was prepared at 36 million cells/mL. Then, 129 μL of GelMA stock was mixed with 43 μL of hMSC suspension; then three 40 μL aliquots of the GelMA/cell mixture were transferred to 1 mL syringes and cured at 365 nm for 3 min. The hydrogels were dispensed into 24-well plates containing 1 mL of basal media per well and allowed to incubate at 37 °C and 5% CO_2_ overnight. The final composition of the hydrogels was 7.5% (w/v) GelMA, 9 million cells/mL, 2 mM LAP, and BMP2 peptide = 0 mM for basal and osteogenic media controls, 0.06, 0.6, or 6 mM. On day 0, the basal media were replaced for osteogenic media, with the media replaced with fresh osteogenic media on days: 3, 6, 9, and 12. The cells were harvested on day 14 for the ALP activity analysis.

### 4.10 ALP Activity Assay

The hydrogels were washed with 1× PBS (3×) and then transferred into 2 mL Eppendorf tubes. To each gel was added 500 μL of ALP lysis buffer containing 1 mM MgCl_2_, 20 μM ZnCl_2,_ and 0.1% (w/v) octyl-β-glucopyranoside in 10 mM tris(hydroxymethyl)aminomethane buffer (pH = 7.4). The hydrogels were homogenized using TissueLyser beads and the TissueLyser II at a frequency of 15 s^–1^ for 5 min. The samples were stored at −80 °C before analysis. A standard curve from 10–800 μM was prepared using 4-nitrophenol in an alkaline buffer. The frozen samples were thawed on ice, transferred to 1.5 mL Eppendorf tubes, sonicated for 10 s, and then centrifuged at 10 000 rpm for 5 min to pellet the cell debris. A sample buffer was prepared by dissolving one 4-nitrophenol phosphate substrate tablet per 3.75 mL of an alkaline buffer solution. Then, in a 96-well plate, 10 μL of supernatant from each sample was mixed with 90 μL of sample buffer in triplicate. The standards and samples were incubated for 30–60 min, then 100 μL of 1 M sodium hydroxide solution was added to terminate the reaction. The absorbance was measured at 405 nm, with the concentration of 4-nitrophenol in the samples determined from the standard curve.

### 4.11 PicoGreen Assay

A 1× TE buffer working solution was prepared. A standard curve from 2000 to 0.02 ng/mL of DNA standard was prepared in 1× TE buffer. A working solution of PicoGreen reagent was then prepared by diluting the stock solution 1:200 (v/v) with 1× TE buffer. Samples were diluted 1:10 (v/v) in 1× TE buffer. Then, a 100 μL aliquot of standard or sample solution and 100 μL of PicoGreen working solution was added to each well of a 96-well plate. The plate was shaken for 5 min, and then the fluorescence was measured using an excitation wavelength = 485 nm and emission wavelength = 525 nm.

## Supporting Information Available

Experimental procedures, Figures S1–S8, Tables S1–S4 (PDF)
