# Supplementary material for: A Facile Method to Quantify Synthetic Peptide Concentrations on Biomaterials
Source: ACS Appl Mater Interfaces. 2024 Sep 9;16(37):49880–8. doi: 10.1021/acsami.4c07164 (PMC11420862; doi:10.1021/acsami.4c07164)
Supplement: Supplementary file 1 — am4c07164_si_001.pdf [file am4c07164_si_001.pdf]

Supporting Information

A Facile Method to Quantify Synthetic Peptide Concentrations on Biomaterials

Jonathan P. Wojciechowski,[1,2‡] *Thomas Benge,*[1‡] *Kaili Chen,*[1] *Cécile Echalier,*[1] *Ruoxiao Xie,*[1] *Molly M. Stevens*[1,2]*

[‡]These authors contributed equally to this manuscript.

[1]Department of Materials, Department of Bioengineering and Institute of Biomedical Engineering, Imperial College London, London, SW7 2AZ, UK.

[2]Kavli Institute for Nanoscience Discovery, Department of Physiology, Anatomy and Genetics, Department of Engineering Science, University of Oxford, Oxford, OX1 3QU, UK.

*molly.stevens@dpag.ox.ac.uk

Table of Contents

Experimental Procedures

Fmoc-Lys(methacrylamide) synthesis S1
LC-MS S4
Peptide synthesis S4
Fmoc-FLAG Peptide LC-MS S7
Fmoc-MA-BMP2 Peptide LC-MS S8
MA-BMP2 Peptide LC-MS S9
Fmoc deprotection in the presence of scavengers S10
Determining Fmoc-Arg(Pbf)-OH loading on H-Rink amide ChemMatrix® resin S10
Gel Permeation Chromatography (GPC) S11
Figure S1 .... S12
Figure S2 .... S13
Figure S3 .... S14
Figure S4 .... S15
Table S1 .... S16
Figure S5 .... S17
Table S2 .... S17
Figure S6 .... S18
Table S3 .... S18
Figure S7 .... S19
Table S4 .... S19
Figure S8 .... S20
References S21

Experimental Procedures

Fmoc-Lys(methacrylamide) synthesis

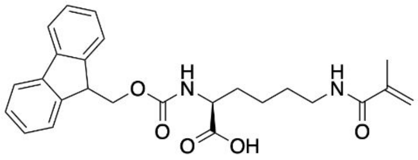

To a 50 mL round bottom flask was added Fmoc-Lys(Boc) (5 g, 10.7 mmol, 1 eq) and a magnetic stirrer bar. Then, a 20 mL mixture containing 1:1 (v/v) trifluoroacetic acid (TFA)/dichloromethane (DCM) and 1 mL of deionized (DI) water was added. The reaction was allowed to stir at room temperature for 2 hours. The TFA/DCM mixture was then removed *via* rotary evaporation before the crude residue was resuspended in DCM and removed *via* rotary evaporation twice. The crude, Fmoc-Lys was then used in the next step without purification.

Fmoc-Lys-COOH was suspended in anhydrous DCM (20 mL). The flask was cooled in a sodium chloride ice bath, then *N*,*N*-diisopropylethylamine (3.72 mL, 21.4 mmol, 2 eq) was added dropwise. A small aliquot of the reaction mixture was removed with a Pasteur pipette, mixed with water, and the pH tested. If the pH was below pH 8, an additional 1 mL of *N*,*N*-diisopropylethylamine was added. This was repeated until pH was greater than 8. The flask was then sealed under nitrogen, then methacryloyl chloride (1.25 mL, 12.8 mmol, 1.25 eq) (was added dropwise. The reaction flask was covered with aluminium foil to protect the reaction from light and allowed to stir overnight and warm slowly to room temperature. The reaction mixture was then diluted with DI water (20 mL) and transferred to a separating funnel. The organic layer was washed with DI water (2 × 20 mL) and brine (20 mL), then dried with anhydrous sodium sulphate, filtered and concentrated via rotary evaporation. The crude mixture was then diluted into HPLC grade 30% acetonitrile in water (v/v) containing 0.1% (v/v) formic acid before being purified using reverse phase preparative high performance liquid chromatography (prep-HPLC) using the gradient below. Eluted fractions were collected based on UV absorbance measurements at 220 and 254 nm with pure Fmoc-Lys(methacrylamide) fractions confirmed using liquid chromatography/mass spectroscopy (LC-MS).

Prep-HPLC gradient profile for Fmoc-Lys(methacrylamide)

Time (min)	B%
0	30
5	30
20	95
25	95
25.5	30
30	30

^{1}H NMR (400 MHz, DMSO-*d*$_6$) δ 7.88 (d, *J* = 7.4 Hz, 3H), 7.73 (d, *J* = 7.5 Hz, 2H), 7.61 (d, *J* = 8.1 Hz, 1H), 7.41 (t, *J* = 7.4 Hz, 2H), 7.33 (t, *J* = 7.4 Hz, 2H), 5.63 (s, 1H), 5.29 (s, 1H), 4.34 – 4.17 (m, 3H), 3.93 (td, *J* = 8.7, 4.5 Hz, 1H), 3.18 – 3.03 (m, 2H), 1.85 (s, 3H), 1.77 – 1.56 (m, 2H), 1.52 – 1.25 (m, 4H). **^{13}C NMR** (101 MHz, DMSO-*d*$_6$) δ 174.05, 167.44, 156.22, 143.89, 143.84, 140.76, 140.15, 127.67, 127.10, 125.32, 120.13, 118.73, 65.65, 53.82, 46.70, 38.62, 30.48, 28.66, 23.11, 18.70. **LRMS** (ESI) calculated for $C_{25}H_{29}N_2O_5$ $[M+H]^+$ 437.2, observed 437.2.

NMR Spectra – Fmoc-Lys(methacrylamide)

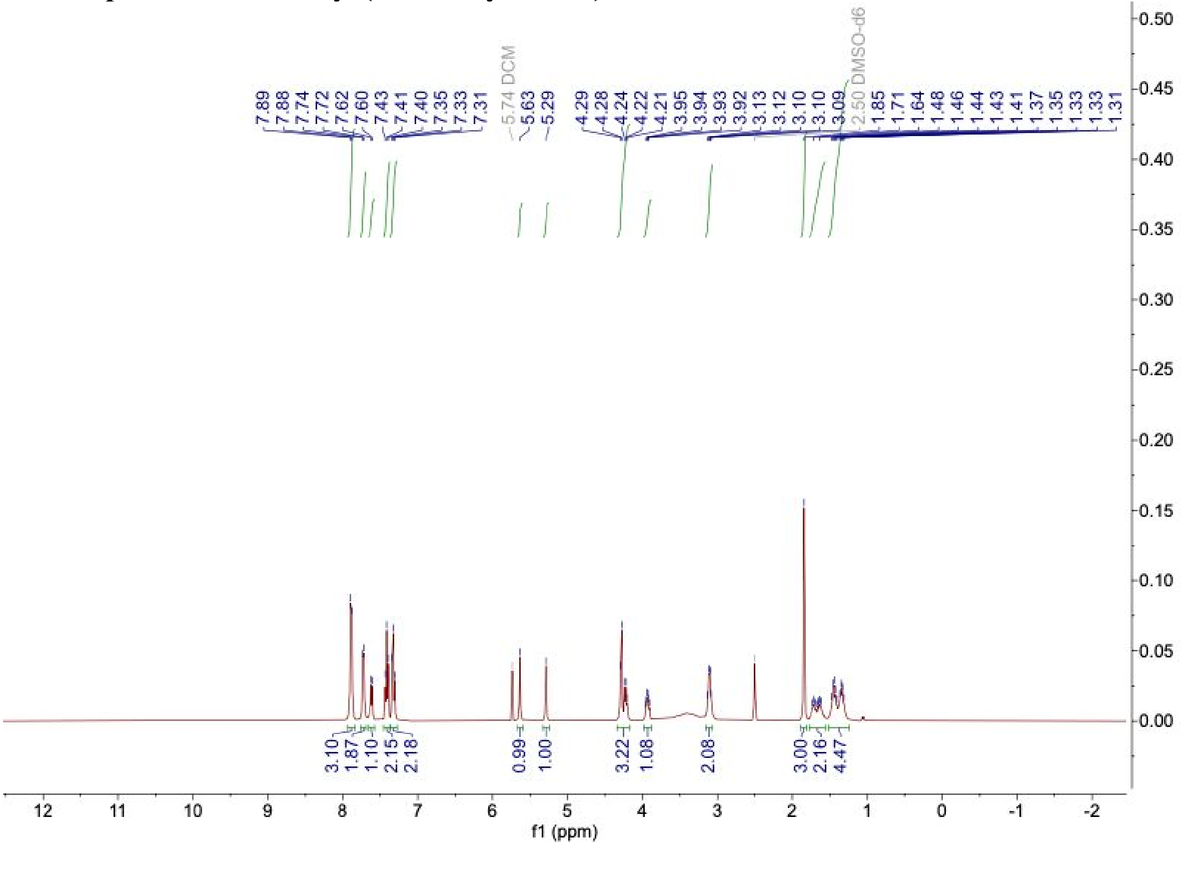

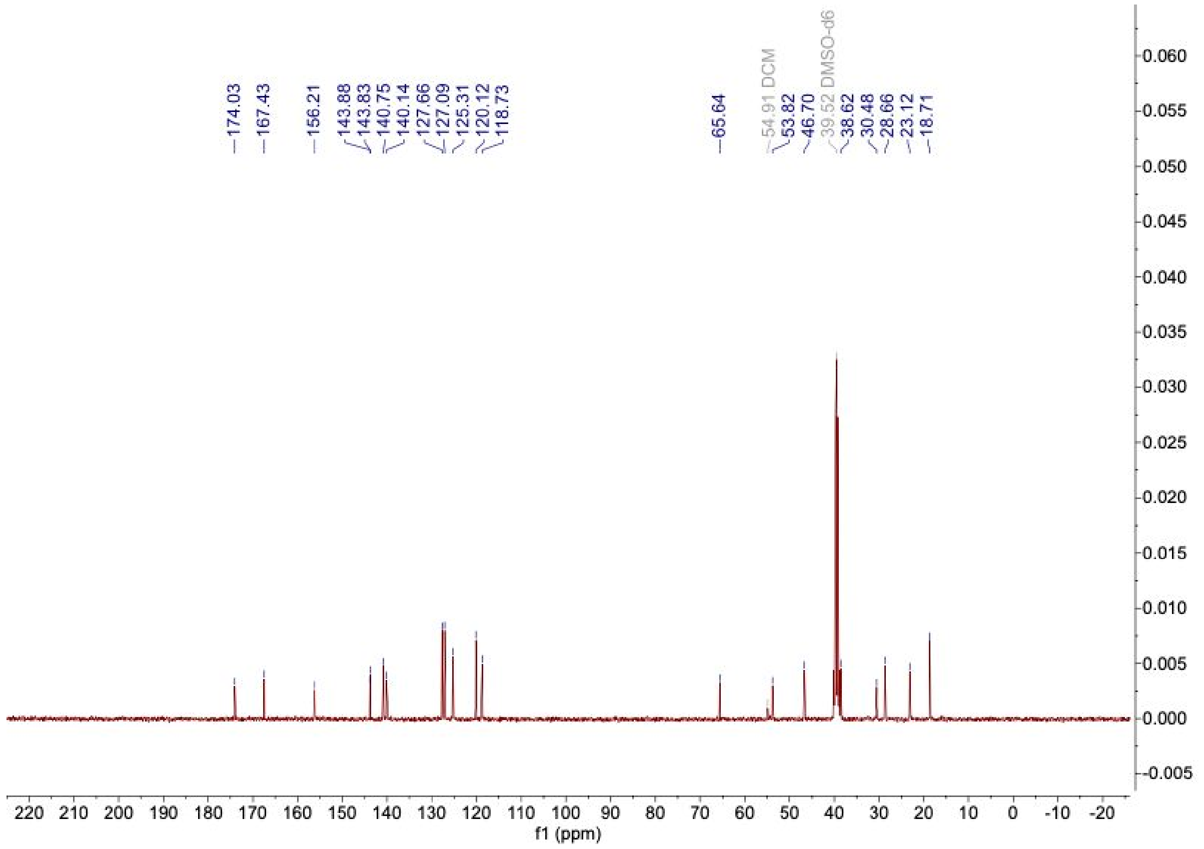
174.03
167.43
156.21
143.88
143.83
140.75
140.14
127.66
127.09
125.31
120.12
118.73
65.64
54.91 DCM
53.82
46.70
39.52 DMSO-d6
38.62
30.48
28.66
23.12
18.71
f1 (ppm)

LC-MS

All LC-MS analysis of the Fmoc deprotections was carried out on an Agilent 1260 Infinity Quaternary LC equipped with an autosampler (G1329A), 1260 Infinity II Diode Array Detector (G7115A), and coupled to an Agilent single quadrupole ESI-MS (G6130B). Measurements were run using a Gemini Phenomenex NX-C18, 50 × 2.1 mm, particle size = 5 µm, pore size = 110 Å, at a flow rate of 0.5 mL/min and injection volume = 5 µL. Mobile phases: A = HPLC grade water with 0.1% (v/v) formic acid, and B = HPLC grade acetonitrile with 0.1% (v/v) formic acid. The gradient profile was as described below:

Time (min)	B%
0	5
0.5	5
4.5	95
6	95
6.1	5
10	5

Peptide synthesis

Fmoc FLAG peptide (Fmoc-DYKDDDDKGGGGC), Fmoc-MA-BMP2 (Fmoc-K(methacrylamide)GGGGKIPKASSVPTELSAISTLYL) and the MA-BMP2 (K(methacrylamide)GGGGKIPKASSVPTELSAISTLYL) peptides were synthesized using a 2-chlorotrityl chloride resin (1.14 mmol/g loading) using standard Fmoc solid phase peptide synthesis conditions. The general procedure is described below.

First-amino acid loading to the resin

To a 12 mL syringe fitted with a polypropylene frit is added 2-chlorotrityl chloride resin (0.5 g, 1.14 mmol/g loading). The resin is washed with dichloromethane (3 × 5 mL), then allowed to swell in dichloromethane (5 mL) on an orbital shaker (200 rpm) for 15 minutes. Meanwhile, a solution containing Fmoc-Cys(Trt)-OH (3 eq) in 1:1 (v/v) *N*,*N*-dimethylformamide/dichloromethane is prepared, followed by the addition of

N,*N*-diisopropylethylamine (6 eq). The dichloromethane solution is expelled from the resin, then the Fmoc-Cys(Trt)-OH mixture is added to the resin. The resin is shaken on an orbital shaker for 16 hours at room temperature, then the solution is expelled. The resin is washed with dichloromethane (5 × 5 mL), then a mixture containing 9:1:0.5 (v/v) dichloromethane/methanol/*N*,*N*-diisopropylethyl amine is added to the resin, which is shaken on an orbital shaker (200 rpm) for 30 minutes. The solution is expelled, then the resin is wash with dichloromethane (3 × 5 mL) and *N*,*N*-dimethylformamide (3 × 5 mL).

Fmoc deprotections

To the resin is added 20% piperidine in *N*,*N*-dimethylformamide (5 mL), which is then shaken on an orbital shaker (200 rpm) for 1 minute then expelled. This step is repeated, but for 15 minutes. The resin is then washed with *N*,*N*-dimethylformamide (5 × 5 mL).

Fmoc-amino acid couplings

A mixture containing the next protected Fmoc amino acid to be coupled (3 e) is prepared by dissolving in a 0.5 M HATU in *N*,*N*-dimethylformamide solution (3 eq). Then, *N*,*N*-diisopropylethylamine (6 eq) is added to this mixture, which is stirred or sonicated to dissolve the Fmoc-amino acid. This mixture is then added to the resin, which is shaken on a orbital shaker (200 rpm) for 30 minutes. The solution is expelled, then the resin washed with *N*,*N*-dimethylformamide (5 × 5 mL). A Kaiser test is performed to determine if the reaction has gone to completion. If a positive Kaiser test result is obtained (*i.e.*, blue-purple coloured solution or resin), this coupling step is repeated.

Cleavage from the resin and global deprotection

The resin is washed with dichloromethane (5 × 5 mL). The syringe plunger is removed then a ST/NS 19/22 glass gas inlet adapter is attached to the back of the syringe. A gentle flow of nitrogen is passed through the resin for 5 minutes to dry the resin. The syringe plunger is the reattached and a cleavage solution (5 mL) containing 2.5% 1,2-ethanedithiol, 2.5% triisopropylsilane, 2.5% milliQ H_2O and 92.5% trifluoroacetic acid (v/v) is added to the resin. The resin is shaken on the orbital shaker (200 rpm) for 3 hours. The solution is then

expelled into a round bottom flask (50 mL), rinsed with trifluoroacetic acid (3 × 2 mL) and concentrated using rotary evaporation. Diethyl ether (45 mL) is added to the concentrated peptide to give a precipitate. The diethyl ether is decanted, and the precipitate then dissolved in 25% acetonitrile in HPLC grade water containing 0.1% (v/v) formic acid. Purification of the peptide was performed using reverse phase preparative HPLC (Shimadzu Prominence LC-20A) equipped with a Phenomenex Gemini® NX-C18 column (150 × 21.2 mm, particle size = 5 μm and pore size = 110 Å) at a flow rate of 15 mL/min, injector loop = 20 mL, and injection volume of 15 mL. Mobile phases: A = HPLC grade water with 0.1% (v/v) formic acid, and B = HPLC grade acetonitrile with 0.1% (v/v) formic acid. The gradient profile was as described below:

**Time (min)**	**B%**
0	5
5	5
20	95
25	95
25.5	5
30	5

Elution fractions were collected based on UV absorbance measurements at 220 and 254 nm with pure peptide fractions confirmed using liquid chromatography/mass spectroscopy (LCMS). Pure fractions were then combined and lyophilized.

Fmoc-FLAG Peptide LC-MS

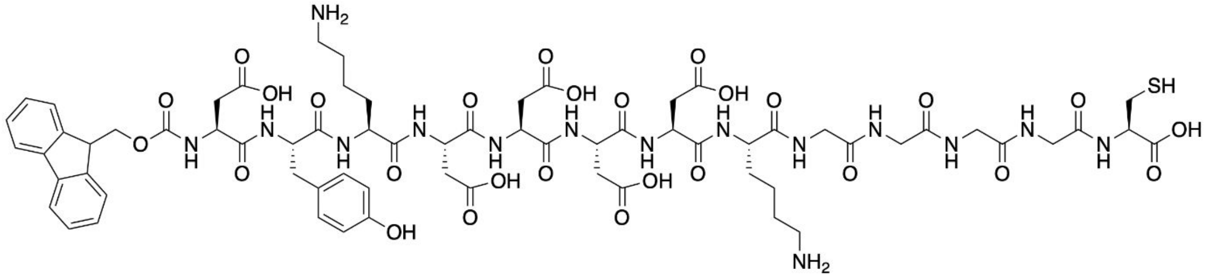

LRMS (ESI) calculated for $C_{134}H_{174}N_{30}O_{54}S_2$ (oxidised, disulfide) $[M+2H]^+$ 1566.1, observed 1566.0 (100); calculated for $C_{67}H_{88}N_{15}O_{27}S$ $[M+2H]^{2+}$ 783.8, observed 783.7 (65).

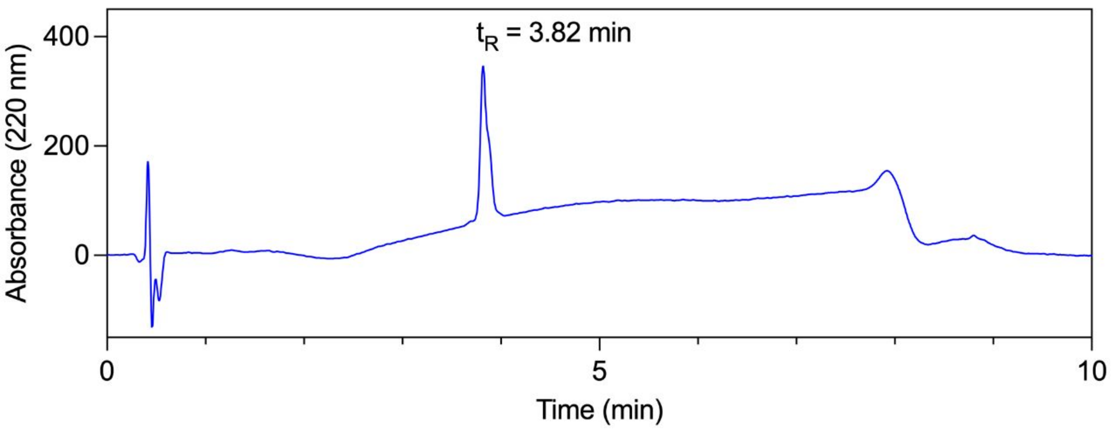

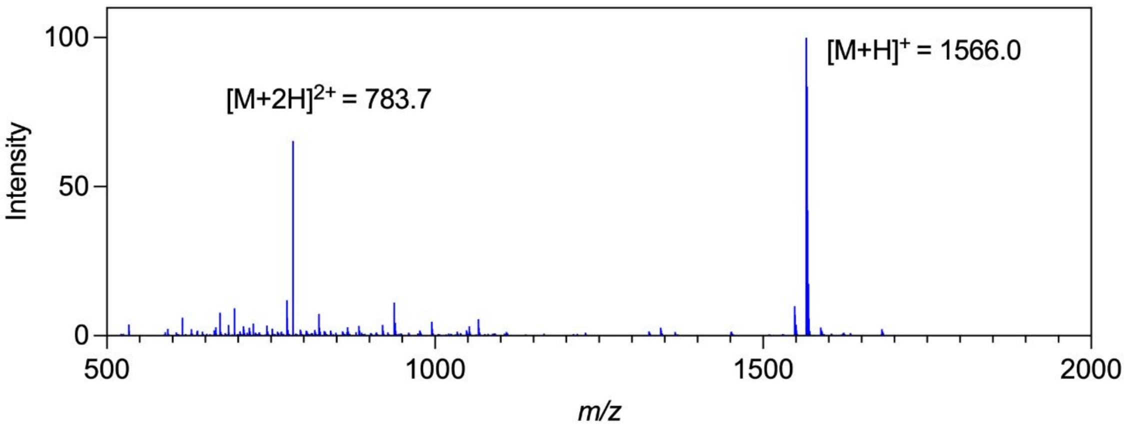

Fmoc-MA-BMP2 Peptide LC-MS

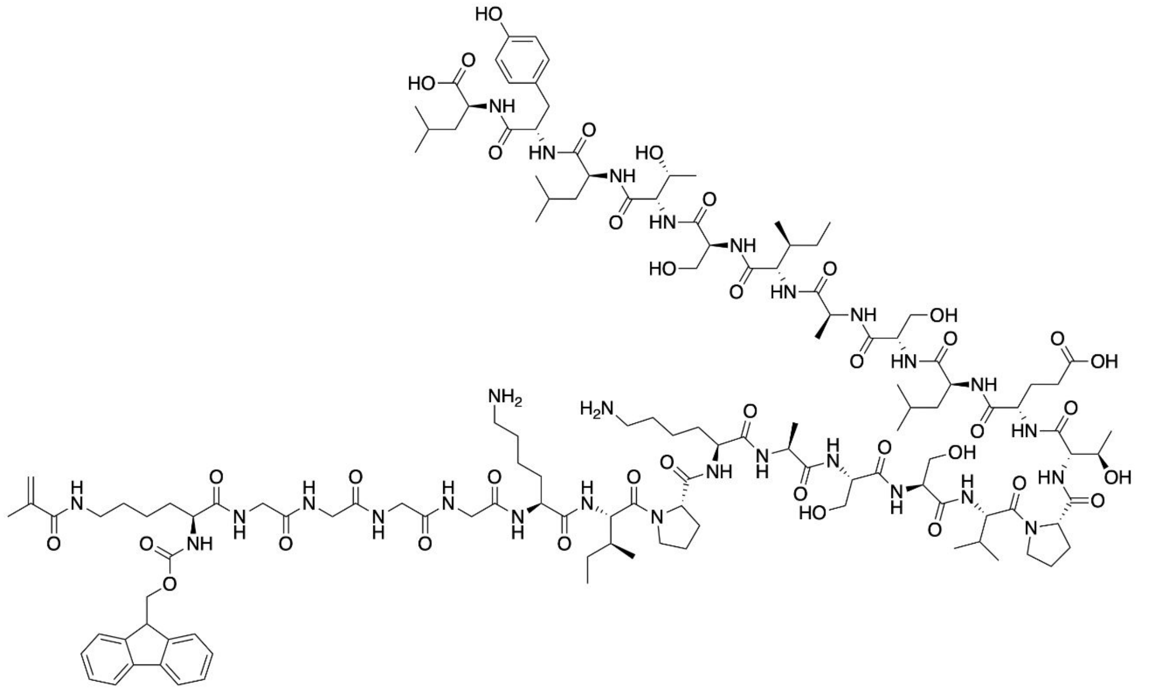

LRMS (ESI) calculated for $C_{130}H_{203}N_{28}O_{38}$ $[M+H]^{+}$ 2764.5, observed 2764.6 (3); calculated for $C_{130}H_{204}N_{28}O_{38}$ $[M+2H]^{2+}$ 1382.7, observed 1382.9 (100).

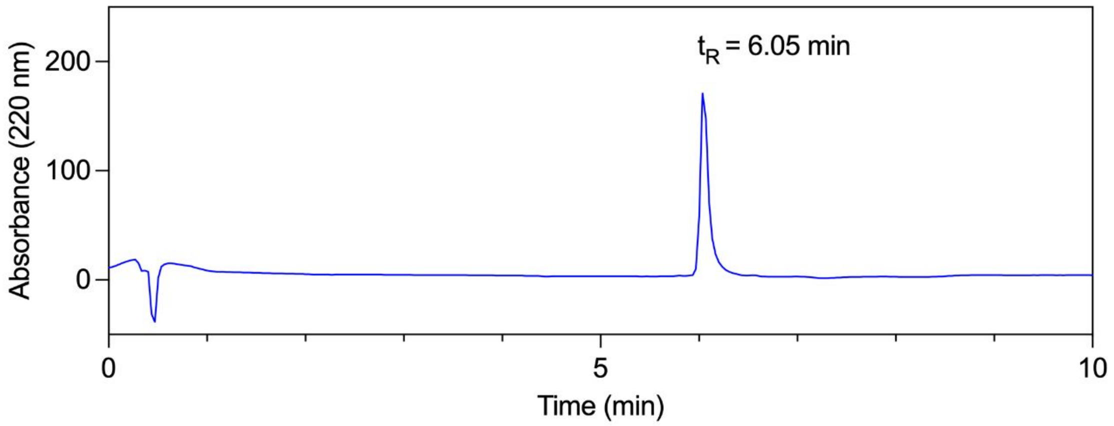

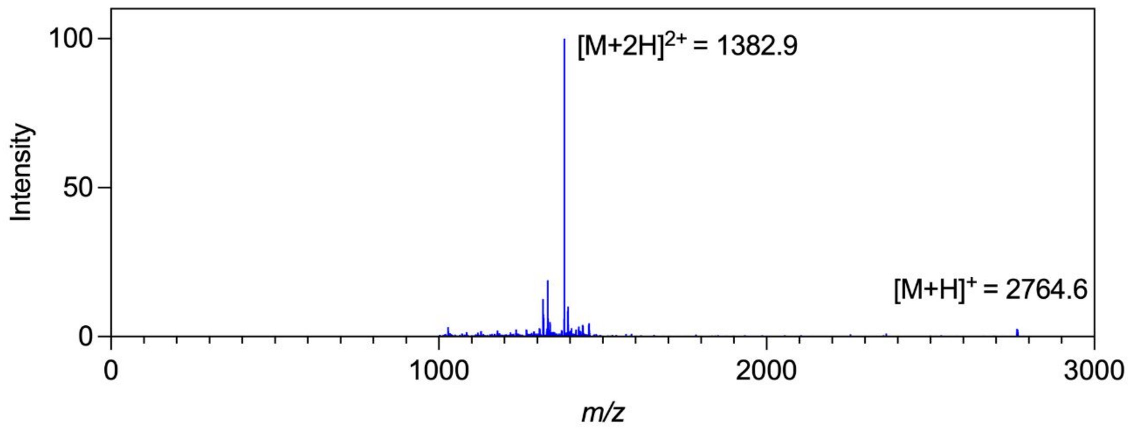

MA-BMP2 Peptide LC-MS

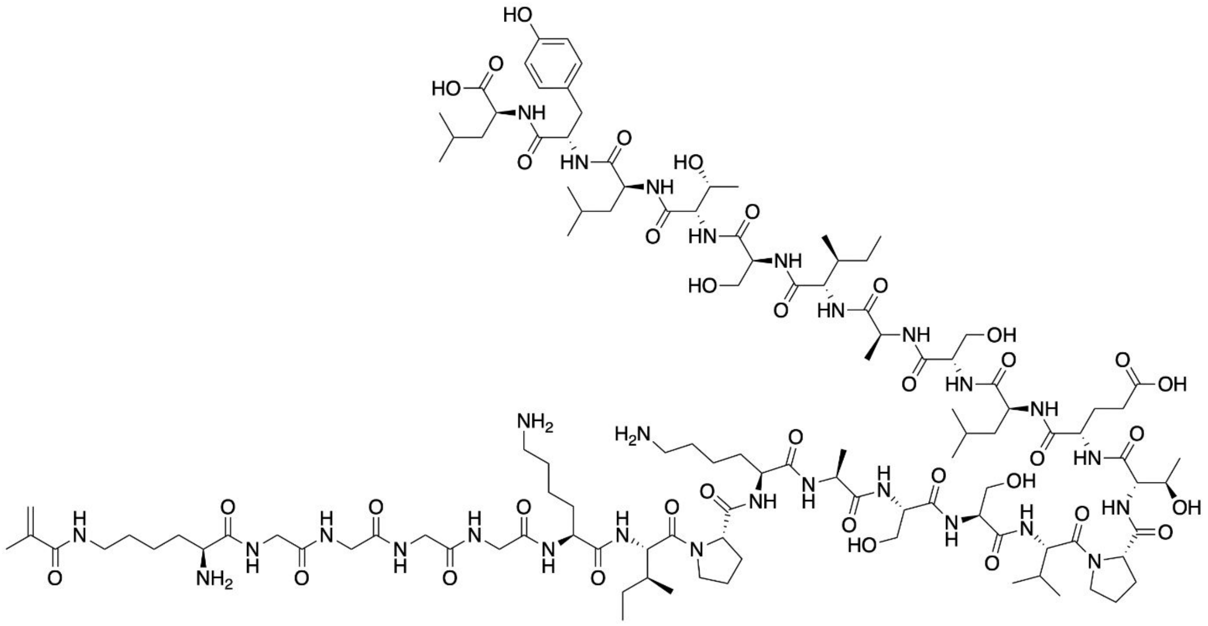

LRMS (ESI) calculated for $C_{115}H_{193}N_{28}O_{36}$ $[M+H]^+$ 2542.4, observed 2542.6 (7); calculated for $C_{115}H_{194}N_{28}O_{36}$ $[M+2H]^{2+}$ 1271.7, observed 1271.9 (100).

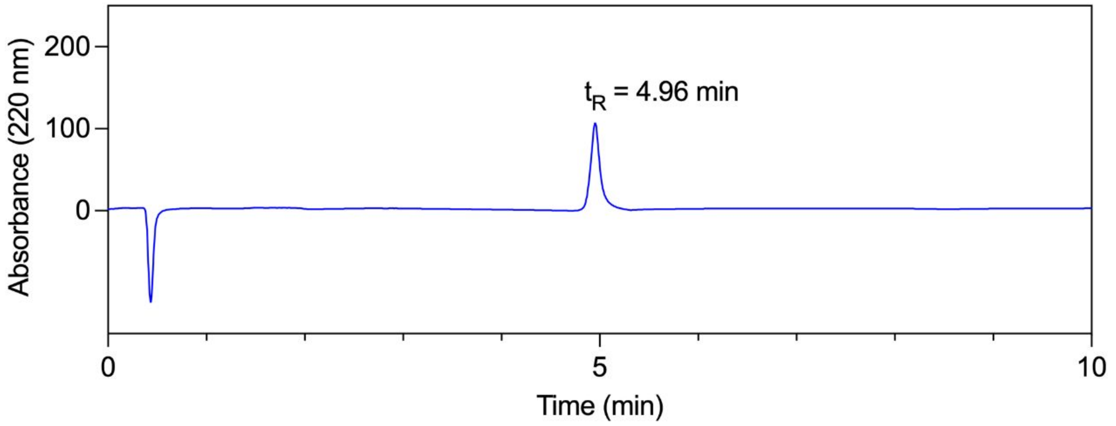

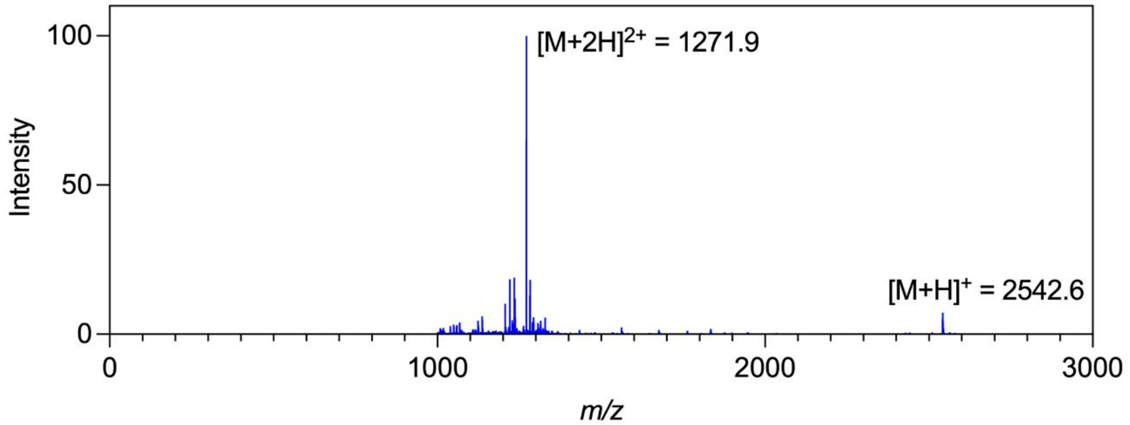

Fmoc deprotection in the presence of scavengers

A 1 mM stock solution of Fmoc-Arg(Pbf) was prepared in 7.5 mL of methanol. A second stock solution containing 100 mM of the scavenger was prepared in 7.5 mL of 0.5 M sodium hydroxide solution. The two stock solutions were combined in equal volumes, and allowed to react at room temperature. An aliquot was removed for HPLC analysis as reaction times of 30 minutes and 24 hours. Assuming comparable extinction coefficients, the ratio of scavenger and the dibenzofulvene peak areas were compared and plotted in **Table S1**.

Determining Fmoc-Arg(Pbf)-OH loading on H-Rink amide ChemMatrix® resin

The loading of Fmoc-Arg(Pbf)-OH was compared using Fmoc-Leu as a reference in 20% piperidine in *N*,*N*-dimethylformamide and the Fmoc cleavage conditions described in this work. In a typical procedure, H-Rink amide ChemMatrix® (150 mg) was washed with dichloromethane (3×5 mL), then left to swell in dichloromethane (5 mL) for 15 minutes. Meanwhile, a coupling solution containing Fmoc-Arg(Pbf)-OH (3 eq), 2,6-lutidine (6 eq) and HATU (3 eq) was prepared in *N*,*N*-dimethylformamide (3 mL). The dichloromethane solution was expelled from the resin, then the coupling solution was added, and left to shake overnight at room temperature. The coupling solution was expelled, the resin washed with dichloromethane (5×5 mL) and *N*,*N*-dimethylformamide (5×5 mL), then a capping solution containing acetic anhydride (3 mL) and pyridine (2 mL) was added, and allowed to react with the resin for 30 minutes at room temperature. The capping solution was expelled, the resin washed with dichloromethane (5×5 mL), then dried under vacuum. For Fmoc-cleavage approximately 10 mg of resin, 3 mg of Fmoc-Leu-OH and 3 mg of Fmoc-Arg(Pbf) was weighed and recorded in separate vials. To the resin, Fmoc-Leu-OH and Fmoc-Arg(Pbf) was added either 1 mL of 20% piperidine in *N*,*N*-dimethylformamide or 0.25 M NaOH in 1:1 (v/v) methanol/water. The samples were shaken for 15 minutes in the case of 20% piperidine in *N*,*N*-dimethylformamide or 30 minutes for 0.25 M NaOH in 1:1 (v/v) methanol/water. After the respective cleavage times, 40 µL of cleavage solution was diluted into 5960 µL of absolute ethanol and measured using a Nanodrop™ 2000c UV-Vis spectrometer. UV-Vis samples were blanked against a blank solution containing 40 µL of 20% piperidine in *N*,*N*-dimethylformamide in 5960 µL of absolute ethanol or 40 µL of 0.25 M NaOH in 1:1 (v/v) methanol/water in 5960 µL of absolute ethanol respectively. The loading of Fmoc-Arg(Pbf) was determined using the extinction coefficient (**Figure S5**) of dibenzofulvene (DBF) and

following procedures described by Al Musaimi *et al* against Fmoc-Leu and Fmoc(Pbf)-OH standards.[1]

The loading of the resin was determined using the extinction coefficient of DBF using:

$$Loading(Resin) = \frac{Abs(294\,nm) \times DilutionFactor}{\varepsilon \times pathlength \times Resin\,mass}$$

The loading of the resin against Fmoc-Leu-OH and Fmoc-Arg(Pbf)-OH standard was determined using:

$$Loading(Resin) = \frac{Abs(301\,nm) \times Standard\,mass\ \times 1000}{Standard\,Abs \times Resin\,mass \times Standard\,M.W.}$$

Gel Permeation Chromatography (GPC).

Polymer molecular weight (Mn,GPC) and dispersity (Đ) were measured using an Agilent 1260 Infinity II GPC MDS (refractive index detection only) equipped with a PSS GRAM guard column (8 x 50 mm, 10 µm) and two PSS GRAM linear columns (8 x 300 mm, 10 µm, 500-1 000 000 Da) The eluent was HPLC grade DMF containing 0.075% (w/v) LiBr and running at a flow rate of 1 mL min-1 at 40 °C. Molecular weight calibration was performed using near-monodisperse poly(methyl methacrylate) standards (EasiVial, Agilent).

Poly(caprolactone), M_w = 80 kDa and poly(lactic acid-*co*-glycolic acid), Resomer RG 756 S, lactide:glycolide 75:25, M_w = 76 – 115 kDa were used as model poly(esters). In a typical experiment, approximately 10 mg of poly(ester) was exposed to 1 mL of 0.25 M NaOH in 1:1 methanol/water for 30 minutes. The solution was removed, the polymer washed 5 × 1 mL with 1:1 methanol/water, dried with a gentle stream of nitrogen, then dissolved in DMF containing 0.075% (w/v) LiBr. The samples were then analysed using GPC as described above.

Supplementary Figures

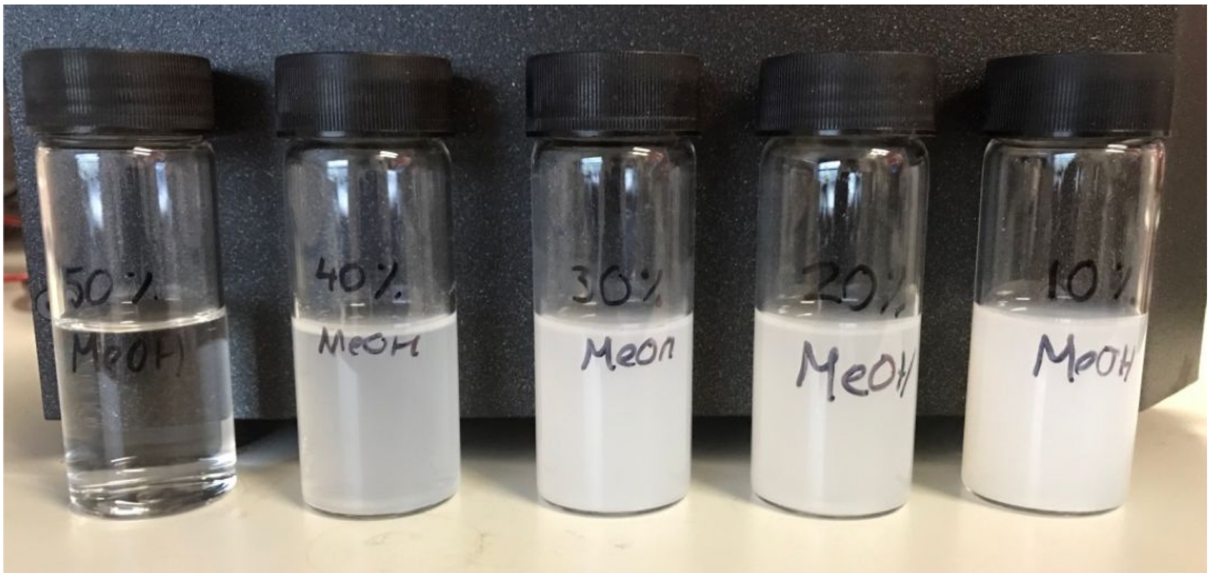

Figure S1. Photo of Fmoc-Arg(Pbf)-OH at 1 mM in methanol/water mixtures (10-50%, v/v) immediately after the addition of aqueous sodium hydroxide (final concentration = 0.25 M). The mixtures became turbid when the concentration of methanol was less than 50% (v/v).

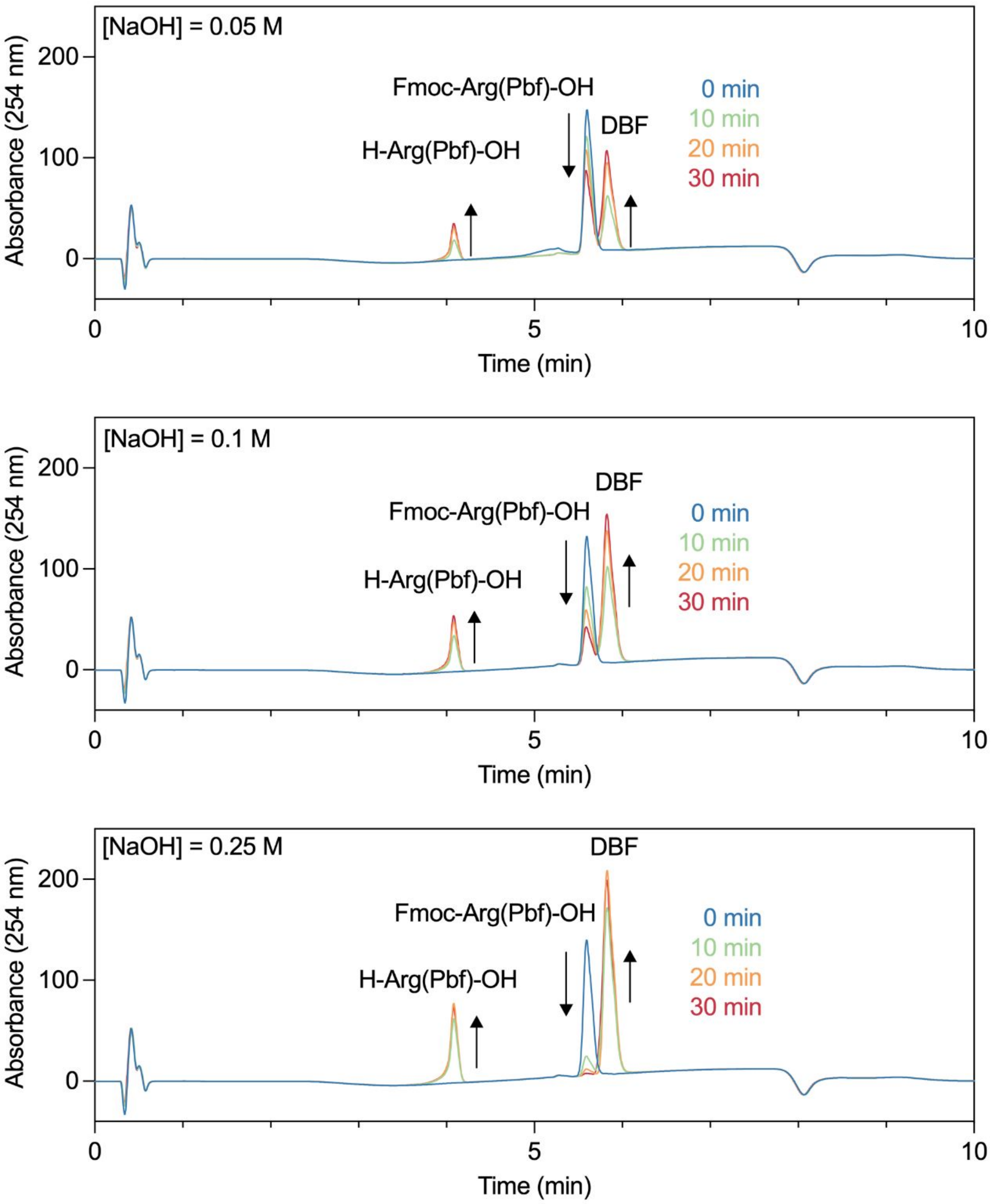

Figure S2. HPLC chromatograms of Fmoc-Arg(Pbf)-OH in various concentrations of aqueous sodium hydroxide (0.05 – 0.25 M) in 1:1 (v/v) methanol/water.

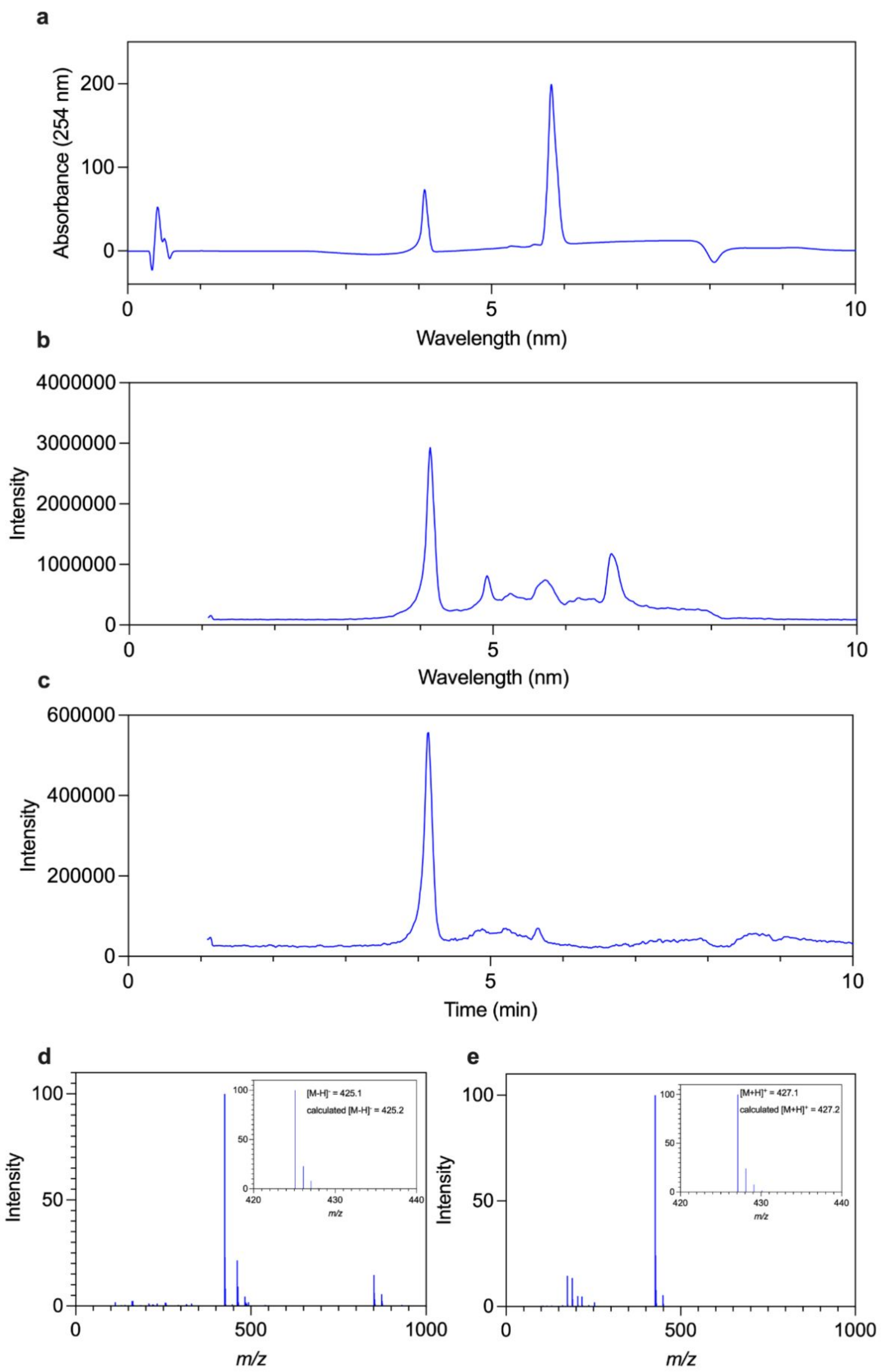

Figure S3. LC-MS characterisation of H-Arg(Pbf)-OH (t_r = 4.08 min). **a** HPLC chromatogram at 254 nm, **b** positive mode ESI-MS TIC, **c** negative mode ESI-MS TIC, **d** positive mode ESI-MS showing $[M+H]^+$ for H-Arg(Pbf)-OH and **e** negative mode ESI-MS showing $[M-H]^-$ for H-Arg(Pbf)-OH.

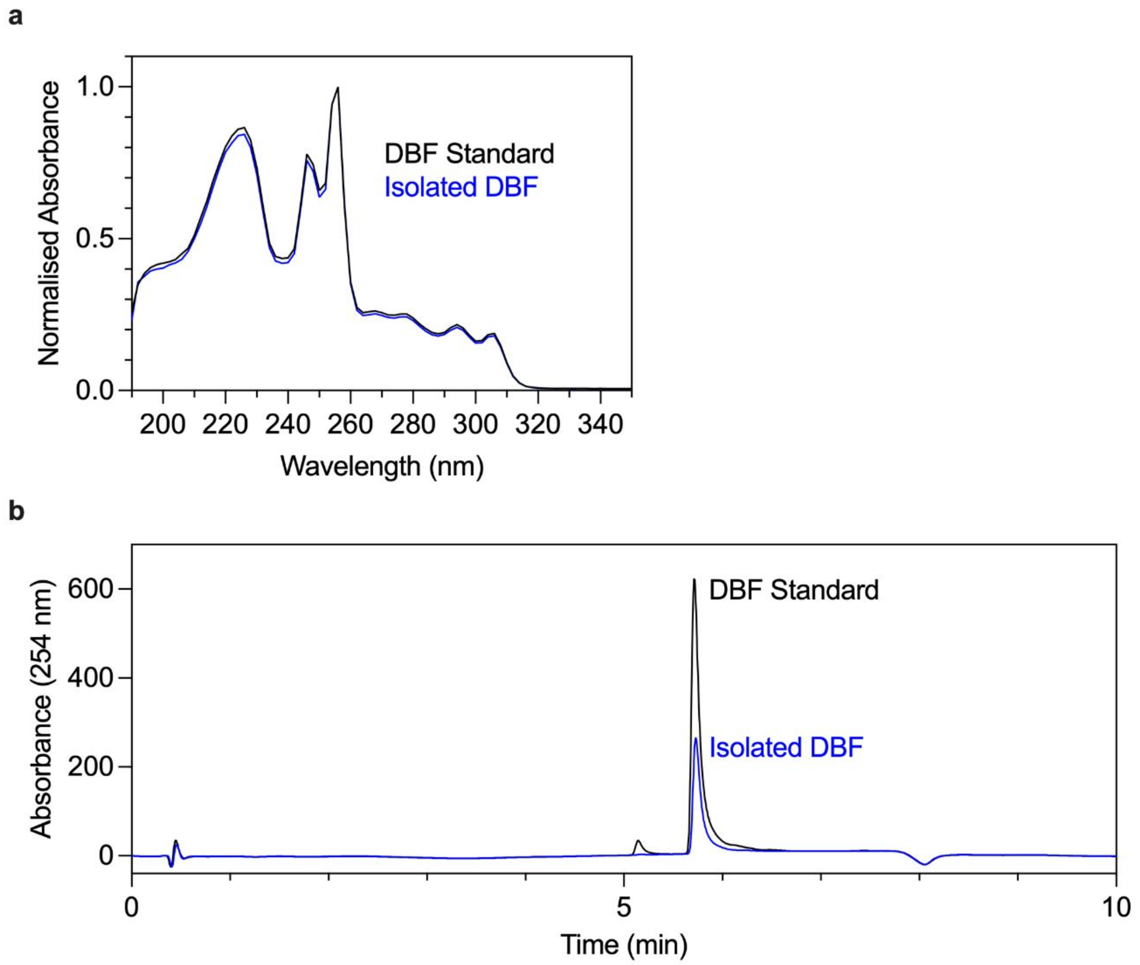

Figure S4. Characterisation of products from the Fmoc-deprotection. The product from the Fmoc deprotect of Fmoc-Arg(Pbf)-OH was purified via preparative HPLC. **a** The UV-Vis spectra of the isolated Fmoc deprotection product shows good agreement against a DBF standard and **b** an identical retention time to the DBF standard.

Table S1. Scavengers used in the Fmoc deprotection to react with the generated DBF.

Scavenger	Molar Equivalents	DBF scavenged after 30 mins	DBF scavenged after 24 hours
**Primary Amines**			
L-Aspartic acid	100	< 0.1%	< 0.1%
L-Glutamic acid	100	< 0.1%	< 0.1%
L-Lysine	100	< 0.1%	< 0.1%
tert-Butylamine	100	< 0.1%	< 0.1%
tris(2-Aminoethyl)amine	100	< 0.1%	< 0.1%
**Secondary Amines**			
L-Proline	100	< 0.1%	< 0.1%
Morpholine	100	2.0%	8.1%
Piperidine	100	Precipitate	Precipitate
Nipecotic acid	100	3.3%	2.9%
Diethylamine	100	< 0.1%	8.3%
1-(2-Hydroxyethyl) piperazine	100	< 0.1%	5.8%
**Thiols**			
L-Cysteine	100	< 0.1%	< 0.1%
D-Thiomalic acid	100	< 0.1%	< 0.1%

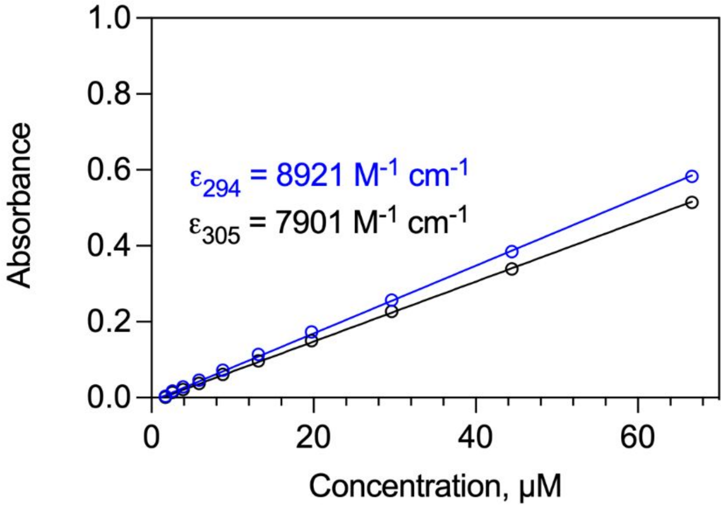

Figure S5. Dilution series of dibenzofulvene (DBF) in 1:1 (v/v) methanol/water. The extinction coefficient is determined from the gradient a plot of absorbance against concentration using Beer-Lambert Law, extinction coefficient, ε (M^{-1} cm^{-1}) = absorbance × pathlength (cm) × concentration (M).

Table S2. Determining Fmoc-Arg(Pbf) on H-Rink amide ChemMatrix Resin

	**20% Piperidine in *N,N*-dimethylformamide**	**0.25 M NaOH in 1:1 (v/v) methanol/H_2O**
Resin Loading using DBF extinction coefficient (mmol/g)	0.213	0.273
Resin Loading using Fmoc-Arg(Pbf)-OH standard (mmol/g)	0.251	0.261
Resin Loading using Fmoc-Leu-OH standard (mmol/g)	0.236	0.257

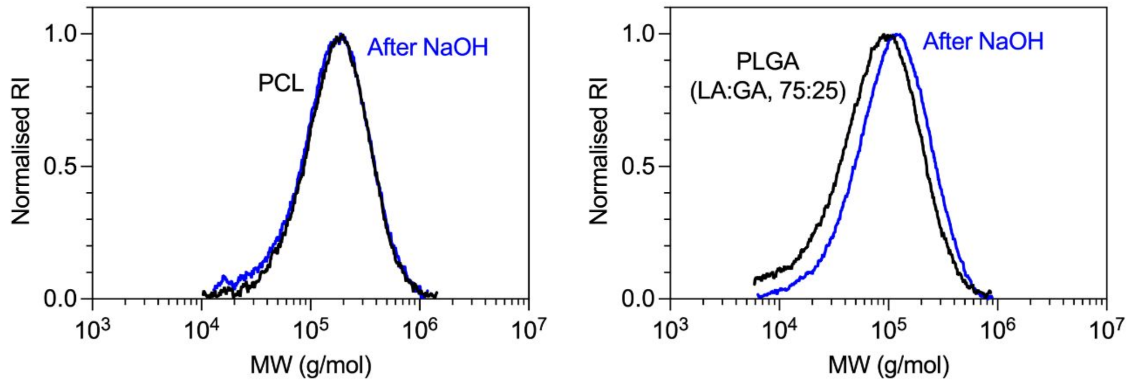

Figure S6. Gel permeation chromatography (GPC) of poly(caprolactone) (PCL), Mw = 80 kDa and poly(lactic acid-*co*-glycolic acid) (PLGA), Mw = 76 – 115 kDa (LA:GA, 75:25) before and after exposure to the Fmoc cleavage conditions.

Table S3. Ratios of components for the synthesis of the Fmoc-FLAG functionalised gold nanoclusters

Molar Ratio Peptide:Glutathione	DI Water (µL)	20 mM $HAuCl_4$ (µL)	2 mM Fmoc-FLAG (µL)	20 mM Glutathione (µL)
1:30	706.5	100	48.4	145.2
1:20	685.7	100	71.4	142.9
1:15	665.6	100	93.8	140.6
1:10	627.3	100	136.4	136.4
1:5	525	100	250	125

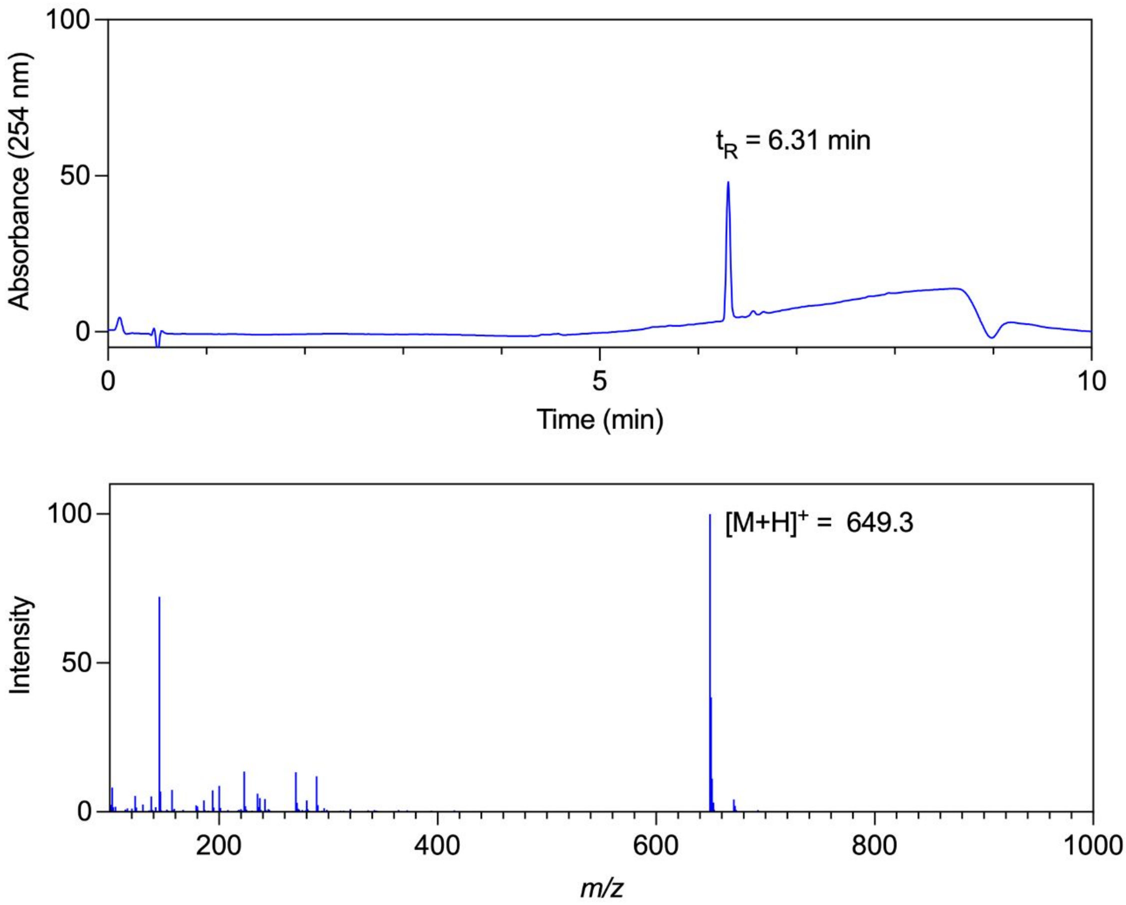

Figure S7. LC-MS of Fmoc-Arg(Pbf) after heating at 70 ºC in DI water for 24 hours. No deprotection of the Fmoc group is observed under the reaction conditions.

Table S4. Ratios of components for the methacrylamide-BMP-2 peptide functionalised GelMA hydrogels

Hydrogel	GelMA (mg)	Concentration of BMP-2 stock (mM)	BMP2 Peptide (µL)	1xPBS (µL)	LAP (µL)
Basal	13.75	N/A	N/A	183	28.1
Osteogenic media	14.05	N/A	N/A	151.7	23.3
2 mM BMP-2	17.18	3.08	119	N/A	18.3
0.2 mM BMP-2	21.2	0.308	122	N/A	18.7
0.02 mM BMP-2	17.5	0.0308	149	N/A	23

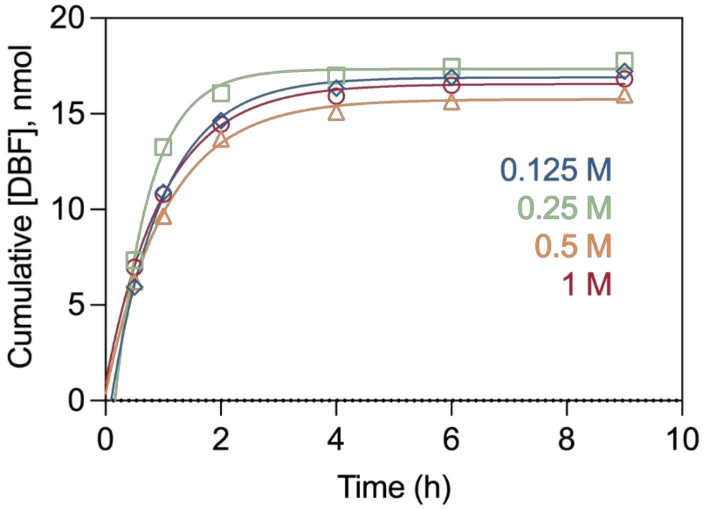

Figure S8. Cumulative released of DBF from 8-Arm PEG hydrogels at various concentrations of aqueous sodium hydroxide (0.125 – 1 M) in 50% (v/v) methanol:water.

References

(1) Al Musaimi, O.; Basso, A.; de la Torre, B. G.; Albericio, F. Calculating Resin Functionalization in Solid-Phase Peptide Synthesis Using a Standardized Method based on Fmoc Determination. *ACS Comb. Sci.* **2019**, *21* (11), 717 – 721. https://doi.org/10.1021/acscombsci.9b00154
